# Neuronal differentiation influences progenitor arrangement in the vertebrate neuroepithelium

**DOI:** 10.1242/dev.176297

**Published:** 2019-12-04

**Authors:** Pilar Guerrero, Ruben Perez-Carrasco, Marcin Zagorski, David Page, Anna Kicheva, James Briscoe, Karen M. Page

**Affiliations:** 1 Department of Mathematics, University College London, Gower Street, London WC1E 6BT, UK; 2 IST Austria, Am Campus 1, A - 3400 Klosterneuburg, Austria; 3 Myrtle Software, Second Floor, 50 St. Andrew's Street, Cambridge CB2 3AH, UK; 4 The Francis Crick Institute, 1 Midland Road, London NW1 1AT, UK

**Keywords:** Vertex model, Neural tube, Computational modelling, Tissue mechanics, Epithelial mechanics

## Abstract

Cell division, movement and differentiation contribute to pattern formation in developing tissues. This is the case in the vertebrate neural tube, in which neurons differentiate in a characteristic pattern from a highly dynamic proliferating pseudostratified epithelium. To investigate how progenitor proliferation and differentiation affect cell arrangement and growth of the neural tube, we used experimental measurements to develop a mechanical model of the apical surface of the neuroepithelium that incorporates the effect of interkinetic nuclear movement and spatially varying rates of neuronal differentiation. Simulations predict that tissue growth and the shape of lineage-related clones of cells differ with the rate of differentiation. Growth is isotropic in regions of high differentiation, but dorsoventrally biased in regions of low differentiation. This is consistent with experimental observations. The absence of directional signalling in the simulations indicates that global mechanical constraints are sufficient to explain the observed differences in anisotropy. This provides insight into how the tissue growth rate affects cell dynamics and growth anisotropy and opens up possibilities to study the coupling between mechanics, pattern formation and growth in the neural tube.

## Abstract (precis)

**Summary:** A mechanical model of the vertebrate neuroepithelium, based on experimental observations, suggests that the rate of neuronal differentiation influences tissue growth and the shape of lineage-related clones.

## INTRODUCTION

The mechanisms that control the arrangement of cells in developing tissues involve both molecular and mechanical processes that spatially and temporally coordinate the division, shape, displacement and differentiation of cells. A central challenge is to understand the interplay between tissue growth, pattern formation and the mechanical forces that act to shape tissues during development.

Studies of several systems have begun to provide insight into how these processes are coordinated (Alt et al., 2017; Merkel and Manning, 2017). For example, in the *Drosophila* wing imaginal disc a combination of experimental observations, quantitative image analysis and computational modelling have revealed the global patterns of mechanical tension that affect the final size and shape of the wing. These patterns result from spatial differences in proliferation, cell shape, division orientation and exchange of neighbouring cells (Shraiman, 2005; Aegerter-Wilmsen et al., 2010; Aigouy et al., 2010; LeGoff et al., 2013; Mao et al., 2013; Guirao et al., 2015; Kursawe et al., 2015; Dye et al., 2017), as well as external mechanical constraints, such as the attachment of the wing blade to the contracting wing hinge (Aigouy et al., 2010; Sugimura and Ishihara, 2013; Etournay et al., 2015; Ray et al., 2015). Molecularly, wing morphogenesis is influenced by planar-polarity signalling, which influences the apical geometry of cells and the orientation of cell division (Aigouy et al., 2010; Mao et al., 2011).

Similar to imaginal discs, the vertebrate neural tube is a pseudostratified epithelium. During neurulation the neuroepithelium folds at the ventral midline and closes dorsally to form a cylindrical neural tube, with the apical surfaces of neural progenitors facing the interior lumen (Gilbert, 2014). The proliferation of neural progenitors contributes to growth of the neural tube along the anterioposterior (AP) and dorsoventral (DV) axes. In addition, proliferating cells undergo interkinetic nuclear movement (IKNM), during which the nucleus of each cell translocates along the apicobasal axis in synchrony with cell cycle progression (Sauer, 1935). A direct consequence of IKNM is that the apicobasal shape, the apical surface of cells and the interactions between neighbouring cells change in a highly dynamic manner (reviewed by Strzyz et al., 2016).

At the same time as the neural tube grows, long-range signals control patterning by regulating the expression of transcription factors within the tissue (reviewed by Sagner et al., 2018). The dynamics of this regulatory network results in the specification of molecularly distinct domains of progenitor subtypes arranged along the DV axis. Each progenitor domain gives rise to a distinct subtype of postmitotic neurons. As neurons are formed, they delaminate basally from the epithelium to the forming mantle zone. The delamination of newly born neurons contributes to the morphodynamics of the neuroepithelium, further reshaping the arrangement of cells within the neural tube.

**Fig. 1.**
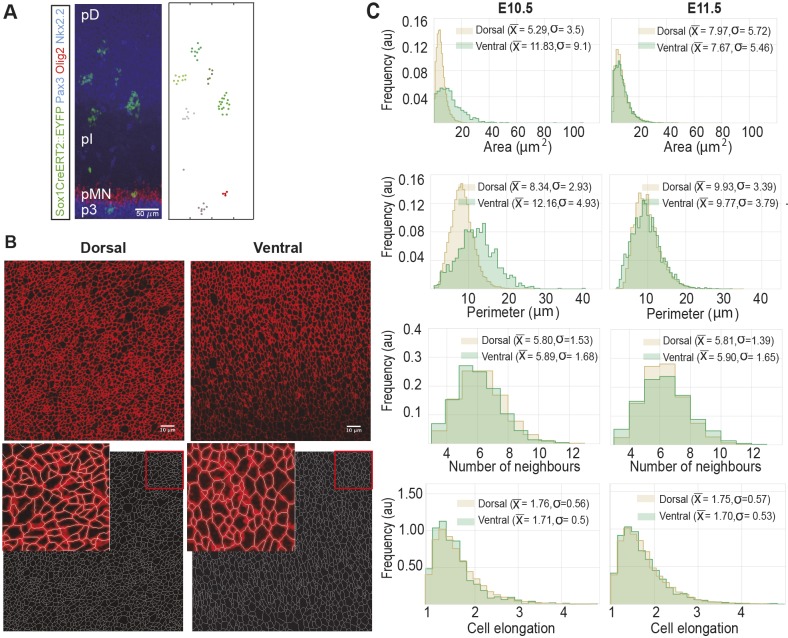
**Analysis of the cellular features of the mouse neuroepithelium.** (A) Example clones in E11.5 embryos, data from Kicheva et al. (2014). Clonal labelling was induced at E9.5 of development. The coordinates of EYFP-labelled cells in the confocal image on the left are shown on the graph on the right. The AP/DV ratio of clones in the pMN domain (red marks) is higher than in the pD domain (green shades). Scale bar: 50 μm. (B) Top panels show the apical surface of E11.5 flat mounted mouse neural tube immunostained for ZO-1. Images were taken within the ventral (right) and dorsal (left) halves of the neural tube. Dorsal side up. Scale bar: 10 μm. Bottom panels show the segmented images after manual correction. Insets show an overlay of original and segmented image. (C) Histograms of apical area, perimeter, number of neighbours and elongation of cells from the dorsal (brown) and ventral (green) regions of E10.5 and E11.5 neural tubes. Sample sizes: E10.5, *n*=25 images of dorsal and five images of ventral domains from seven different embryos; E11.5, 11 images of dorsal, three images of ventral domains from three embryos.

Previous studies of the neural tube have indicated that patterning and growth are tightly coordinated. Cell death is negligible and the rate of progenitor proliferation is spatially uniform throughout the epithelium (Kicheva et al., 2014). However, the rates of terminal neuronal differentiation vary depending on progenitor identity. Most notably, starting at mouse embryonic day (E)9.5, motor neuron progenitors (pMN) differentiate at a significantly faster rate than other progenitor subtypes (Ericson et al., 1996; Kicheva et al., 2014). This difference in the rates of terminal differentiation correlated with a difference in clone shape in lineage tracing experiments (Kicheva et al., 2014; Fig. 1A). In particular, although the AP spread of clones in all domains was similar, the DV spread was not. Clones in all but the pMN domain were more elongated along the DV axis compared with the AP axis. By contrast, clones in the pMN domain have an average AP/DV ratio of ∼1 indicating equal growth in DV and AP directions. This raises the question of what mechanisms operate to ensure equivalent AP growth across the tissue, while at the same time allowing for cell-type-specific differences in DV growth rates.

To address this, we developed computational tools to simulate the growth of the neuroepithelium and investigate the role of different mechanisms in the morphodynamics of the tissue. We made use of a representation of the apical 2D surface of the epithelium by employing a vertex model formalism (Nagai and Honda, 2001; Farhadifar et al., 2007; Smith et al., 2012; Fletcher et al., 2013). Vertex models have been used successfully to describe mechanical and molecular influences that determine the tissue growth and form of several epithelia (e.g. Farhadifar et al., 2007; Landsberg et al., 2009; Aegerter-Wilmsen et al., 2010; Wartlick et al., 2011; Trichas et al., 2012; Salbreux et al., 2012 and others) and we chose this approach to test whether the experimentally observed variations in clone shape could be explained by the mechanics of the neuroepithelium. In these models each cell is represented as a polygon, the vertices and edges of which are shared between adjacent cells. The dynamics of a cell are described by the movement of its vertices, which are controlled by adhesive/tensile, contractile and repelling forces in and between cells.

To take account of the 3D configuration of the neural tube, we incorporated the effects of IKNM into the simulation framework. Using experimental data from the mouse neural tube, we then established model parameters for which simulations match *in vivo* observations. We used the resulting model to explore clonal shape within the neuroepithelium and the effect of spatially varying the differentiation rate within the tissue. Strikingly, we found that the increased differentiation rate of pMN progenitors is sufficient to explain the different shape of clones within the pMN domain. This indicates that the differences in clonal shape arise from differences in progenitor differentiation rates and global mechanical constraints, and do not require polarised molecular signalling mechanisms. Simulations of the developing neuroepithelium using this model can contribute to our understanding of how tissue patterning and growth are controlled and coordinated.

## RESULTS

### Cell geometry in the mouse neuroepithelium

To construct a mechanical model of neural tube growth we first measured key features of neural progenitor organisation in the mouse embryonic neural tube. To this end, we imaged the apical tight junctions of the neural tube at forelimb level of E10.5 and E11.5 mouse embryos (Fig. 1B, top). The images were segmented, vertices and edges defined using ‘Packing Analyzer v2.0’ (Aigouy et al., 2010) (Materials and Methods; Fig. 1B, bottom).

Images from the dorsal half of the neural tube comprise the progenitors of dorsal interneuron subtypes, and we refer to this region as the pD domain. Images from the ventral half of the neural tube contain motor neuron and intermediate progenitor subtypes and we term this the pMN region (Methods). From the segmented images we determined the distributions of cell areas, cell perimeters, number of neighbours per cell and cell elongation (Fig. 1C). Cells in all samples had on average six neighbours as expected (Graustein, 1931; Classen et al., 2005; Gibson et al., 2006), with a standard deviation of ∼1.5. There were some differences in the mean and variance of cell areas and perimeters in the samples (Fig. 1C), which were most noticeable at E10.5, when the rate of neuronal differentiation is highest in the pMN (Kicheva et al., 2014). Nevertheless, the average area of cells assayed in this way was consistent with previous measurements (Kicheva et al., 2014). Also, consistent with Lewis's Law, the relative average area of cells increases linearly with the number of cell neighbours (Fig. S1; Kokic et al., 2019 preprint). Using these data, we set out to develop an *in silico* model of the neuroepithelium.

### A vertex model of the neuroepithelium including IKNM

We constructed a 2D vertex model of the apical surface of the neural tube in which cells are represented as polygons. The behaviour of each cell is governed by the movement of its vertices that follow a deterministic overdamped motion given the energetic contributions of cell elasticity, junctional forces arising from cortical contractility and the effect of cell-cell adhesion and cortical tension (Kafer et al., 2007; Lecuit and Lenne, 2007; Farhadifar et al., 2007; Hilgenfeldt et al., 2008; Landsberg et al., 2009; Aegerter-Wilmsen et al., 2010; Fletcher et al., 2014; Honda and Nagai, 2015; Alt et al., 2017). For the purposes of the simulation we developed custom Python code using an Euler method to solve the movement equation of each vertex, Eqn 4.

**Fig. 2.**
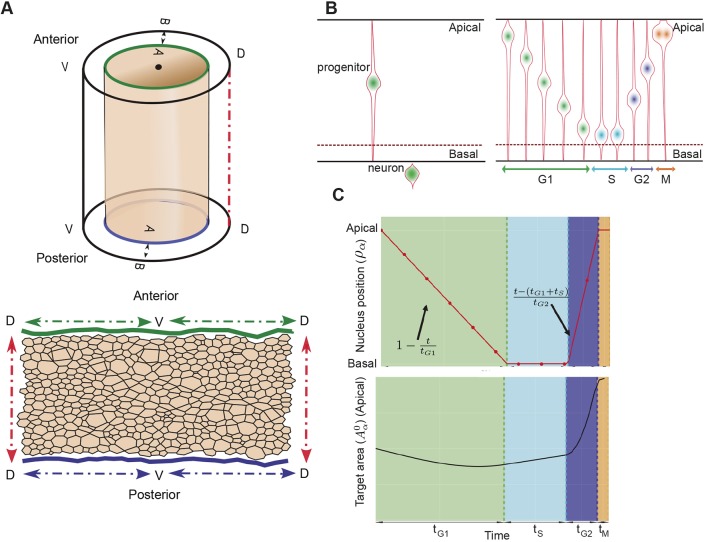
**Vertex model simulation framework for the neural tube incorporating interkinetic nuclear movement.** (A) Top panel shows a 3D diagram illustrating the DV, AP and apical-basal (AB) axes on a cylindrical representation of the neural tube. Bottom panel shows a 2D polygon representation of the apical junctional network. In the simulations, periodic boundary conditions mean that the top-bottom and left-right edges are continuous. (B) Left panel represents the neuronal differentiation of a progenitor leading to loss of the apical contact and cell extrusion from the epithelium. Right panel represents IKNM: the apicobasal position of the nucleus changes with cell cycle phase. The nucleus moves basally during G1, undergoes S-phase basally, returns apically in G2 and is apical during mitosis. (C) Top panel shows a representation of the function used to describe nuclear position (*ρ*_*α*_) during a simulated cell cycle. Bottom panel shows the apical target area that results from Eqn 1 during a cell cycle. Coloured regions in the top and bottom panels indicate different cell-cycle phase durations: *t*_*G*1_, G1-phase (green); *t*_*S*_, S-phase (light blue); *t*_*G*2_, G2-phase (dark blue); *t*_*M*_, M-phase (orange). This is an illustration for an example cell, the exact cell cycle behaviour of individual cells in simulations will vary.

Topologically, the neural tube is a cylinder that grows at different rates along its AP and DV axes (Fig. 2A). Our analysis focuses on a region along the AP axis at the forelimb level. This region is relatively small compared with the entire length of the AP axis and is distant from the influence of the rostral and caudal limits of the neural tube. To take this into account and to avoid artefacts from introducing external boundary conditions, we used periodic boundary conditions in the AP axis by simulating the neural tube as a torus. Growth results in the circumferential and radial increase in the size of the torus over time. For visualisation we unwrapped the torus by cutting along both DV and AP axes to allow simulations to be rendered in 2D (Fig. 2A, bottom).

To describe the behaviour of neural progenitors within the simulation, a detailed description of cell growth, division and differentiation is required. Upon neuronal differentiation, cells lose their apical attachments and are extruded basally from the epithelium (Fig. 2B, left). During each cell cycle, progenitors in the neuroepithelium undergo IKNM, in which their nuclei and the bulk cell volume translocate along the apical-basal axis of the neuroepithelium. Mitosis occurs at the apical surface of the epithelium. Nuclei move basally in G1 and undergo S-phase towards the base of the epithelium. During the G2-phase, nuclei migrate back to the apical surface for mitosis. A consequence of IKNM is that the apical area of cells, corresponding to the surface represented in the simulations, is affected by the cell cycle stage. When cells enter mitosis, they round up at the apical surface. This expands their apical area and compresses neighbouring cells. As a consequence, cells are likely to achieve their largest apical surface area in late G2 and M phase, and their smallest surface area in S-phase. The measured duration of cell cycle phases (Kicheva et al., 2014) (Table 1) can therefore be used to derive an approximation for the temporal changes in apical surface area of cells caused by IKNM.

**Table 1.**
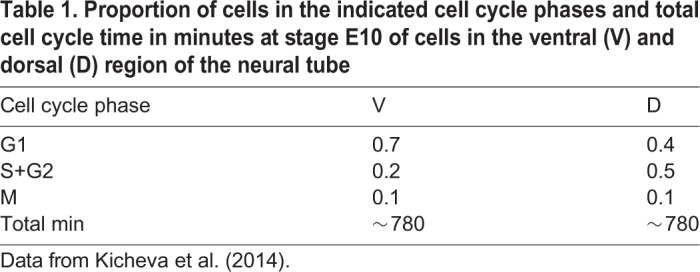
**Proportion of cells in the indicated cell cycle phases and total cell cycle time in minutes at stage E10 of cells in the ventral (V) and dorsal (D) region of the neural tube**

To accommodate the effect of IKNM in our simulations we introduced a time-dependent target area function, *A*_α_^0^(*t*) (Eqn 1), which describes the desired apical area of the cell. This function depends on the age of the cell and the cell cycle phase and was constructed to account for the measured cell cycle dynamics:(1)
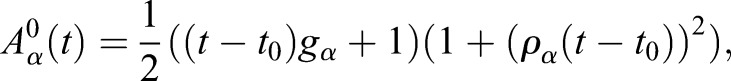
where *g*_*α*_ is the growth rate of the cell *α* [for each cell this is chosen randomly from a normal distribution with a mean equal to the inverse of the life span of a cell (1/780 min) and a variance of 20% of the mean], *t*_0_ is the moment when the cell *α* is born and *ρ*_*α*_(*t*−*t*_0_) represents the apical-basal position and depends on the phase of the cell cycle by a piece-wise linear function incorporating the dynamics of the cell cycle:(2)
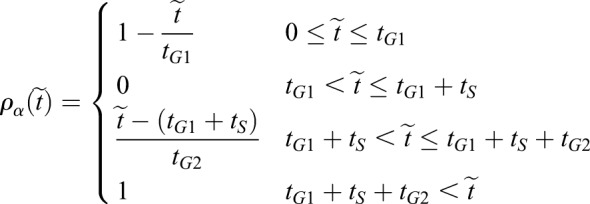


where *t*_*G*1_, *t*_*S*_ and *t*_*G*2_ are the respective cell cycle phase durations and *t̃* = *t* − *t*_0_. The position of a cell body along the apicobasal axis (see scheme in Fig. 2B, right) is given by the function *ρ*_*α*_(*t*) where apical is 1 and basal is 0. The function *ρ*_*α*_(*t*) is defined by four different straight lines which correspond to each cell cycle phase (Fig. 2C). In *G*1, the nuclear movement is from apical to basal and takes *t*_*G*1_ time and thus decreases linearly with time at rate 1−(*t*/*t*_*G*1_). During S-phase, the nucleus stays basal for time *t*_*S*_, and *ρ*_*α*_(*t*) is set to 0. Basal to apical migration occurs during *G*2, over the period *t*_*G*2_, and is represented by the increasing function 
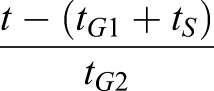
. During mitosis, the function takes value 1. The functional form of *A*_α_^0^(*t*) is the product of a term which grows linearly in time, as we assume the volume of the cell does, and a term which interpolates between 1/2 and 1, as the cell moves from the basal to the apical surface, with a higher rate of increase as it approaches the latter surface.

Implemented in this way, the target area of a cell, which describes the desired apical area of the cell, takes account of both cell growth during the cell cycle and the position of the cell body along the apicobasal axis. It results in the apical area slowly reducing during G1, corresponding to the cell body moving towards the basal surface at the same time as the cell is growing, then increasing slowly during S-phase, rapidly expanding during G2, as the cell returns to the apical surface for division, and growing slowly during mitosis (Fig. 2C, bottom). In simulations we use the dorsal cell cycle phase times, indicated in Table 1, where *t*_*S*_ is 1/3 and *t*_*G*2_ is 2/3 of the S+G2 proportion time. Note that the target area given above is in nondimensional units. For the nondimensionalisation, see Materials and Methods, Vertex model implementation.

Division occurs when the cell is in M-phase (*t̃* > *t*_*G*1_ + *t_S_* + *t*_*G*2_) and the volume of the cell exceeds a critical value, *A*_*c*_. As a cell undergoes division two new vertices are created to form a new edge. One of these vertices is chosen as the midpoint of a randomly selected edge of the dividing cell with probability proportional to the edge length. The other vertex is the midpoint of the opposite edge, and if the cell has an odd number of sides, the second edge is the closer mid edge. The newly generated sister cells then commence the next cell cycle.

In the neural tube, newly generated neurons lose their apical attachments and delaminate basally (Fig. 2B, left). Hence, neuronal differentiation leads to the loss of cells from the plane of the neuroepithelium. In the simulation, this is achieved by identifying cells committing to differentiation, suppressing growth in these cells by assigning their target area equal to zero and allowing their area to decrease. As the area of a cell drops, some of its edges become small and disappear under certain T1 transition conditions (see below), which ultimately results in elimination of the cell. At the stage of development we are modelling, cells differentiate predominantly within the pMN domain. In simulations, we select cells to differentiate with a fixed probability per unit time.

The combined effect of cell growth, division and differentiation results in cells moving relative to each other, producing local remodelling of the epithelium and rearrangements of neighbouring cells. In the simulations, these topological rearrangements occur through T1 transitions. During a T1 transition an edge shorter than a prescribed length (chosen to be 3% of the average edge length in the tissue) is eliminated and a new edge of length *l*_*new*_ expands perpendicular to the old edge (values given in Table 2). However, if the rearrangement results in the formation of a two-sided cell, the cell is removed from the epithelium.

**Table 2.**
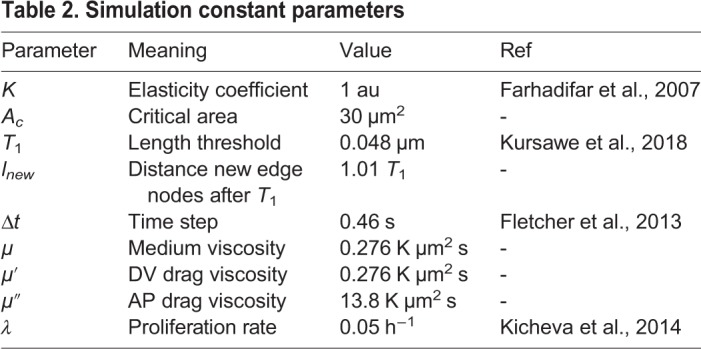
**Simulation constant parameters**

### Simulation parameter estimation

We next used the experimental data to identify model parameters for which simulations match *in vivo* observations. The dynamics of the simulations are determined by the Hamiltonian (Eqn 5) that takes into account the energetic contributions of different cellular mechanical properties. The minima of this Hamiltonian can be described using two dimensionless parameters: 
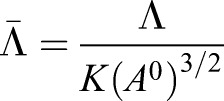
 and 
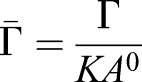
, where *A*^0^ denotes the average of the target area, *A*_α_^0^, during the cell cycle (Farhadifar et al., 2007). This average value was obtained as the mean value of the target area across all cells at the end of 12 simulations and was 1.25 nondimensional units. In the standard implementation of the model this leads to a phase diagram describing four different parameter regions in which the tissue has different biophysical properties, (Supplementary Materials and Methods, section II; Farhadifar et al., 2007; Magno et al., 2015).

Similar to some previous studies (Canela-Xandri et al., 2011; Kursawe et al., 2018), the target area term of the Hamiltonian we used includes a cell cycle dependent component. However, as vertex movement is substantially faster than the cell cycle, the same phase diagram remains applicable (for more details of the derivation of the phase diagram see Supplementary Materials and Methods, section II).

**Fig. 3.**
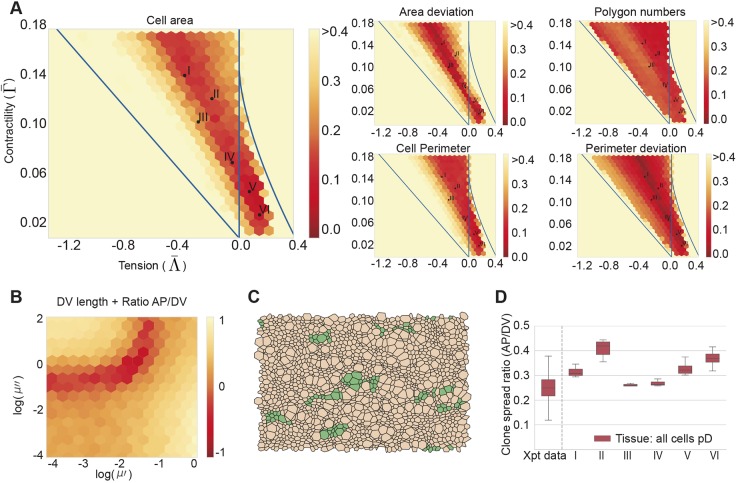
**Identification of simulation parameters matching experimentally determined features of the neuroepithelium.** (A) Similarity of experimental data and simulations with the indicated composite tension and contractility parameters. Colour code represents the average of the maximum distance of the empirical cumulative distribution function for cell area, cell perimeter and polygon number, and the absolute difference between the standard deviations of cell area and cell perimeter (see Materials and Methods). Experimental data is from E11.5 embryos and simulations correspond to 10 independent simulations per point in parameter space. Roman numbered dots indicate the mechanical parameters selected to study clonal distribution, (Λ̄, Γ̅): I, (−0.4, 0.14); II, (−0.2, 0.12); III, (−0.3, 0.1); IV, (−0.05, 0.065); V, (0.075, 0.04); VI, (0.15, 0.02). *μ*′ and *μ*″ are 0.02 and 1, respectively. Blue lines demarcate the four different parameter regions of the phase diagram (see Supplementary Materials and Methods, section II). (B) Heatmap as in A, but the absolute value of the difference (log scale) in the change in DV length of the tissue over 48 h plus the absolute value of the difference in the final tissue aspect ratio (AP/DV) between simulations and experimental data was taken and this quantity was averaged over 10 simulations for each point. (Λ̄, Γ̅) used for these simulations are from the mechanical parameter set V. (C) Examples of the shape of simulated clones tracked over 48 h demonstrate the DV bias in their elongation *in silico*. Brown represents pD cells, green represents clones of pD cells. (D) Comparison of clone spread ratio (AP/DV) between experiments (Xpt data) and simulations. Box plot shows the quartiles of 12 realisations of the simulation with no differentiation (all cells pD domain); median values (middle bars) and interquartile ranges (boxes); whiskers extend from the box to show the range of the data 1.5x the interquartile range past the first and third quartiles. Roman numerals indicate mechanical parameters used (Λ̄, Γ̅) in A. Other parameter values are provided in Table 2.

We focus our attention on the region of the phase diagram exhibiting epithelial properties (Regions II and III in Fig. S10); this is given by the following relation between normalised tension and contractility parameters, (Magno et al., 2015):(3)
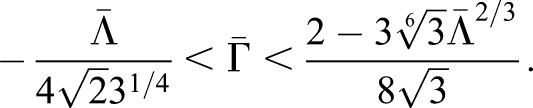
To narrow down the region of parameter space (Λ̄, Γ̅) relevant for neural tube simulations, we systematically screened parameter sets to identify those that generated cell geometries comparable with experimental data. We compared experimental and simulated empirical cumulative distribution functions (ECDF) of area, perimeter, number of cell sides and cell elongation (defined in Materials and Methods, Experimental data analysis) and values of standard deviation of area and perimeter. We used experimental data from E11.5 dorsal neural tubes compared with the ECDF obtained from vertex model simulations with different combinations of Λ̄ and Γ̅. Assessing the match between experimental and simulation data indicated a diagonal region in parameter space for which all the measured features of the *in silico* cell geometries closely matched those observed *in vivo* (Fig. 3A). The shape of this region is similar to previously published vertex model simulations (Kursawe et al., 2018). Moreover, the agreement with experimental data was better in the model with IKNM (Fig. 3A), compared with a standard model formulation without IKNM in which the target area is constant over time (Fig. S2). Therefore, in subsequent simulations we used the model with IKNM and six parameter sets selected from different locations from within the region of parameter space representing the best agreement with experimental data.

### Simulating anisotropic tissue growth

We next turned our attention to the overall tissue growth. Our previous experimental studies (Kicheva et al., 2014) indicated that the tissue grows asymmetrically in DV and AP directions. During the period under consideration, the DV length of the tissue increased more than the AP length. This effect was reflected in the shape of clones of lineage-related cells, such that the mean ratio of AP to DV spread of the clones outside of the pMN domain was ∼0.3 (Kicheva et al., 2014).

In the simulations, expansion along the DV and AP axes is resisted by drag forces that have coefficients *μ*′ and *μ*″, respectively. A difference between these two coefficients generates different rates of DV and AP tissue growth and consequently alters the tissue AP/DV aspect ratio (Figs S3,S9; Supplementary Materials and Methods, section I), imitating the effect of physical constraints on *in vivo* tissue expansion. For all six selected Λ̄, Γ̅ parameter sets, we identified a range of values of *μ*′ and *μ*″ that were consistent with both the experimentally observed AP/DV aspect ratio and reduction of the DV length of the pMN domain (Fig. 3B; Fig. S3A). Within this range of dimensionless values, the values *μ*′∼0.02 and *μ*″∼1 matched closely the AP/DV ratio of 0.3 (Fig. S3B) and were used in further analysis.

To test the effect of these asymmetric forces, we examined the shape of clones in simulations by tracking lineage-related cells *in silico*. For this, simulations were started from a field of 100 cells and run for 30 h of biological time to allow the simulation to equilibrate. Following this initialisation period, the progeny of individual cells were tracked for a further simulated 48 h. This corresponds to an average of 3-4 cell divisions, mimicking the experimental conditions in which the *in vivo* clonal data were generated. Similar to the experimental data, *in silico* lineage-related cells (clones) tended to form coherent groups and the shape of clones was similar between experiments and simulations (Fig. 3C,D). For all six parameter sets, cells within a clone tended to spread more along the DV axis compared with the AP axis to give an *in silico* AP to DV aspect ratio of ∼0.3 (Fig. 3D), similar to clones in the mouse neural tube (Kicheva et al., 2014). Thus, with the identified parameters, there was a good correspondence between the behaviour of cells in the simulation and those in the real neuroepithelium.

### The rate of neuronal differentiation affects the shape of progenitor clones

Clones in the pD domain have a lower AP/DV aspect ratio than clones in the pMN domain. Having established a simulation framework and identified parameters that mimic neuroepithelial behaviour, we set out to address what could account for the difference in clone shape between domains. Progenitors within the pMN differentiate at a substantially higher rate than other progenitors at this stage of development (Kicheva et al., 2014), raising the possibility that this accounts for the difference in clone shape.

**Fig. 4.**
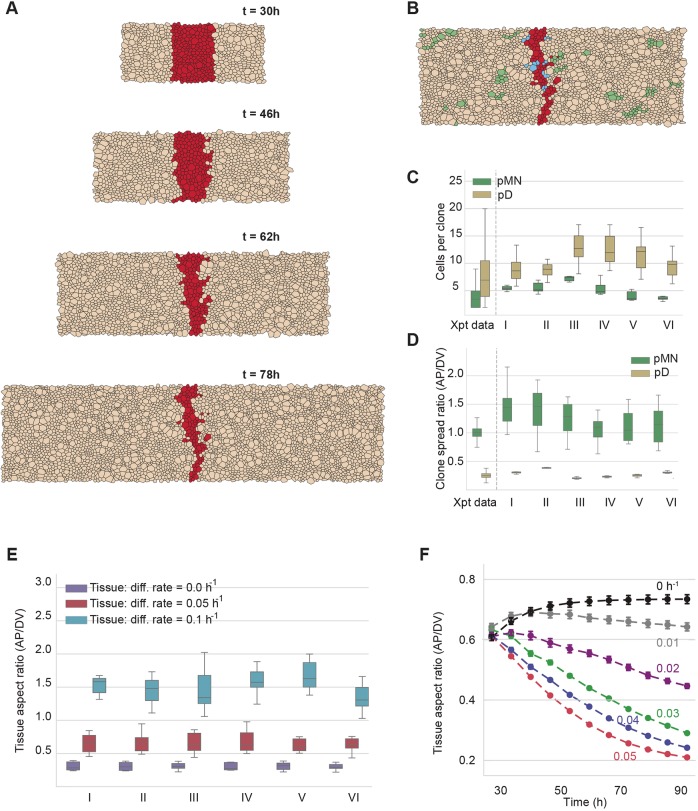
**The rate of neuronal differentiation affects the anisotropy of tissue growth.** (A) Snapshots from a simulation of a tissue with pD (brown) and pMN (red) populations. Differentiation in the pMN domain is initiated at 30 h, after which the relative DV length of the pMN domain decreases from 30% to 5% over 48 h. (B) Examples of clones tracked during the 48 h simulation *in silico*. Brown represents pD cells, green represents clones in pD domain, blue represents clones in pMN domain, red represents pMN cells. (C) Comparison of number of cells per clone between experimental data (Xpt data) and simulations in pMN (green) and pD (brown). (D) The ratio of AP/DV spread of clones in the pD (brown) and pMN domain (green) domains compared with experimental data (Xpt data). (E) Tissue aspect ratio (AP/DV) for simulations of homogeneous tissues consisting of a single cell type with the indicated differentiation rates: 0 h^−1^ (purple), 0.05 h^−1^ (red) and 0.1 h^−1^ (light blue). (F) Tissue aspect ratio (AP/DV) over time of simulated tissues with the indicated proliferation rates and differentiation rate equal to zero for a given regime V. Box plots show median values (middle bars) and interquartile ranges (boxes); whiskers extend from the box to show the range of the data 1.5x the interquartile range past the first and third quartiles. Data in C-F is derived from 12 different simulations per parameter set and Roman numerals indicate the mechanical parameters (Λ̄, Γ̅) from Fig. 3A. Differentiation rate in pMN for A,B,C and D is 0.1 h^−1^. Other parameters are indicated in Table 2.

We implemented a pMN domain in simulations by defining a region of tissue with an appropriate differentiation rate. Following the initialisation period, a pMN domain comprising 30% of DV length of the neural tube was introduced by imposing a differentiation rate of 0.1 h^−1^ on cells in this region, corresponding to the maximum differentiation rate of pMN progenitors *in vivo* (Kicheva et al., 2014). The remainder of the tissue was designated as pD domain and lacked differentiation, representing the more slowly differentiating dorsal progenitor domains *in vivo*. Simulations were continued for a further period equivalent to 48 h of biological time (Fig. 4A).

At the end of the simulations, the proportion of tissue composed of the pMN decreased from the initial 30% DV length of the tissue to ∼5% DV length for all six mechanical parameter regimes (Fig. 4A; Fig. S4A). This is a consequence of the increased differentiation rate resulting in a loss of progenitors from the pMN. This decrease in the DV extent of the pMN matches the experimentally observed reduction in the DV proportion of the neuroepithelium occupied by the pMN domain from 30% of the neural tube at E9 to 5% 48 h later at E11 (Kicheva et al., 2014) (Fig. S4A). Moreover, clones in the pD domain comprised 8-12 cells on average, consistent with an average of 3-4 cell divisions that occur in the 48 h period (Fig. 4B; Movie 1). By contrast, pMN clones contained 4-5 cells per clone. These *in silico* clone sizes are consistent with the clone sizes observed in the experimental data (Fig. 4C). Together these data indicate that the behaviour of the simulated pMN and pD domains matches the behaviour observed *in vivo*.

We then examined the spread of clones along the AP and DV axes (Fig. 4D). Similar to the simulations lacking a pMN domain (Fig. 4D), clones within the pD region were anisotropic with an AP/DV aspect ratio of ∼0.3. By contrast, for all six parameter sets, clones within the simulated pMN domain had a substantially higher AP/DV aspect ratio (*P*<0.05, two-sided *t*-test, Fig. 4D). The marked difference between the AP/DV aspect ratio of the pMN and pD domains was seen in both experiments and simulations, independent of the number of cells in a clone (Fig. S5). These results reveal that the difference in the shape of clones in the pMN compared with the rest of the neural tube can be explained by the increased differentiation rate of these cells.

### The anisotropy of tissue growth depends on the net growth rate

To investigate how the increased rate of differentiation affects the anisotropy of tissue growth, we analysed how tissue anisotropy changed when different differentiation rates (0-0.1 h^−1^) were imposed uniformly throughout the tissue. This revealed that a higher differentiation rate correlated with a larger AP/DV aspect ratio of the tissue (Fig. 4E). This effect is consistent with the observation that a higher differentiation rate correlates with a higher AP/DV aspect ratio of clonal shape (Fig. 4D; Fig. S6). Furthermore, this tendency was present for all ratios of *μ*″/*μ*′ larger than one (Fig. S7). This indicates that as long as there are global anisotropic drag forces, the differentiation rate determines the exact extent of tissue growth anisotropy, with higher differentiation rates yielding higher AP/DV aspect ratios.

The decreased anisotropy of clones of cells in the pMN domain might result from the decreased net growth rate of the pMN, rather than directly from the increased differentiation. To investigate the effect of tissue growth rate on anisotropy, we began by further simplifying the problem and constructing a simple model in which we assumed cells were identical and rectangular (Supplementary Materials and Methods, section III). This predicted that the aspect ratio tends asymptotically to a value that depended on the drag coefficients and the growth rate. For very slow growth of the tissue (low net proliferation rate), the aspect ratio would be close to one, whereas for very rapid growth, it would be close to the square root of the ratio of drag coefficients (Supplementary Materials and Methods, section III). Thus, the effect of the drag on tissue anisotropy would be less pronounced for slow growth rates, leading to more isotropic growth. To test this hypothesis, we ran simulations without differentiation but with varying proliferation rates (Fig. 4F). Consistent with our hypothesis, the slower proliferation rates decreased the anisotropy of tissue growth. Thus, increased differentiation per se was not necessary for the observed behaviour. Instead, the net growth of tissue affects its aspect ratio.

Further investigation showed that the effect of differentiation rate on the aspect ratio of the tissue was more complex than simply slowing the effective tissue growth. Although both increasing the differentiation rate and decreasing the proliferation rate correlated with decreasing tissue growth anisotropy at a given time (Fig. S6A-F), the anisotropy that corresponded to a specific net growth rate was not always the same. Instead, different degrees of anisotropy were achieved for the same net growth rate, depending on whether proliferation or differentiation was modulated (Fig. S6F). Furthermore, tissues of similar size (generated by similar net growth rates) would adopt different aspect ratios depending on the relative contributions of proliferation versus differentiation to the net tissue growth (Fig. S6A-F). Tissues that differentiate have increased anisotropy (lower AP/DV ratio) compared with tissues that reach the same size without differentiation (compare Fig. S6G and S6H). We postulate that increasing differentiation rate facilitates the rearrangement of internal boundaries which allows the tissue to tolerate more tissue growth anisotropy. Consistent with this, a high differentiation rate (0.1 h^−1^) increased the frequency of T1 transitions (to 0.38±0.04 per cell per h by the end of the simulation) compared with simulations with no differentiation (0.14±0.03 per cell per h). Thus, differentiation has opposing effects: it slows growth, which tends to make growth more isotropic (Supplementary Materials and Methods, section III shows that if cells cannot rearrange, the final anisotropy of the tissue increases with the exponential growth rate of cells) and it facilitates internal boundary rearrangements, which tends to allow tissue growth to be more anisotropic (Supplementary Materials and Methods, section III shows that if cells are free to rearrange to become individually isotropic, the total energy is independent of the tissue aspect ratio, which is therefore primarily controlled by the anisotropic drag). Further work will be needed to fully understand the determinants of tissue aspect ratio.

**Fig. 5.**
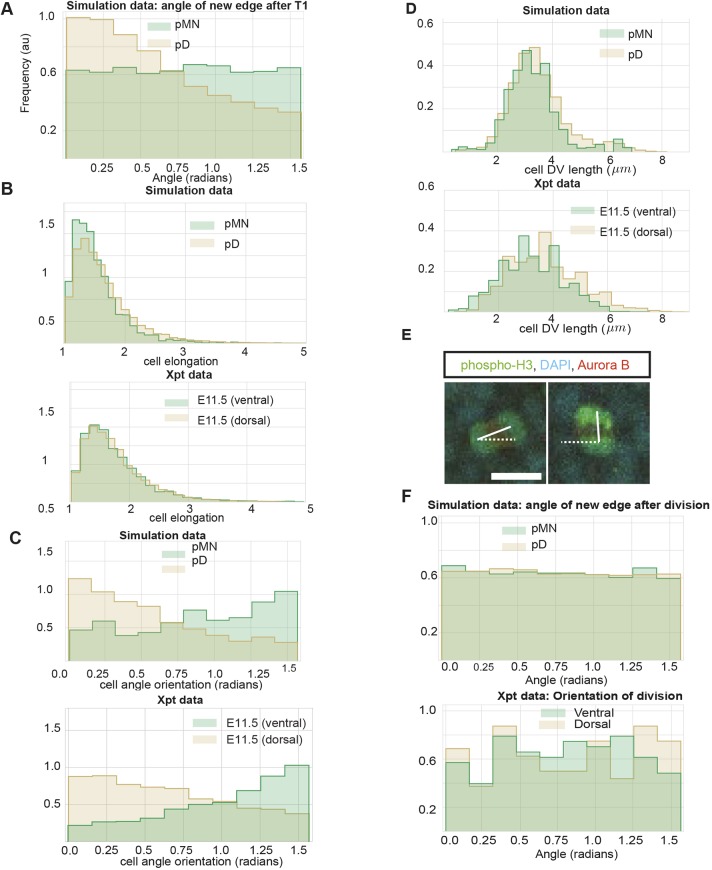
**Contribution of cell rearrangements, cell shape and cell division orientation to anisotropic growth.** (A) Orientation angle with respect to the DV axis of the newly formed edge after a T1 transition in the pMN (green) or pD (brown) domains. (B) Distributions of cell elongation in simulations and experimental data at E11.5. (C) Distributions of the orientation angle of the long axis of cells with respect to the DV axis in simulations and experimental data at E11.5. (D) Cell DV length distribution calculated by taking the vertex coordinates and calculating min(x)-max(x) (*x*-axis represents DV direction) in simulations and experimental data in the indicated regions. (E) The angle of cell division orientation *in vivo* as outlined by the white lines in two example cells was measured at E10.5. Dotted line indicates AP axis. Scale bar: 10 μm. (F) Distributions of cell division orientation in simulations and experimental data in the indicated regions. Angle is represented by radians where 0 is parallel to the DV axis. The angle of the new edge after cell division should be roughly orthogonal to the axis of cell division. For simulations, distributions were estimated after 12 realisations using mechanical parameter regime V. Differentiation rate in pMN domain 0.1 h^−1^. Other parameters are indicated in Table 2. For experimental data, distributions were calculated at E11.5. Eleven images of the dorsal and three images of the ventral domain from three embryos were used in B,C,D and F.

We next turned our attention to the cellular dynamics that result in the anisotropic growth of the tissue. We first measured the orientation of T1 transitions. In the pD domain, the orientation of a T1 transition more frequently resulted in topological rearrangements that replace an AP-directed edge with one in the DV direction (Fig. 5A). Such transitions cause cells to intercalate, expanding the DV axis. There was no such bias in cells of the pMN domain. A consequence of these dynamics was a change in the orientation of cells. Measurements of cell elongation, defined as the square root of the ratio of the eigenvalues of the second moment matrix of the vertices of the cell (Materials and Methods), indicated that cells were equally elongated in the pMN and pD domains (Fig. 5B). However, in the pD domain cells tended to be orientated with their long axis in the DV direction, whereas cells in the pMN domain tended to be orientated with their long axis in the AP direction (Fig. 5C). Strikingly, these differences in cell orientation between cells in the dorsal and ventral halves of the neural tube were also observed in the experimental data (Fig. 5C). This change in cell orientation resulted in a difference of the mean DV length of cells, with pD cells having approximately 10% larger DV lengths than pMN cells in both simulations and experiment (Fig. 5D). Thus, the change in cell shape alone had a minor contribution to anisotropic tissue growth, suggesting that T1-mediated cell rearrangements is the main factor driving the DV extension of the tissue.

In contrast to the bias in cell rearrangements, the axes of cell divisions in our simulations were distributed uniformly in both the pD and pMN domains (Fig. 5E,F) indicating little, if any, bias in division orientation. In the simulations this is a consequence of mitotic cells markedly reducing their elongation, allowing for random orientation of the division angle (Fig. S8). To test whether this was consistent with the *in vivo* observations we examined the orientation of mitotic spindles in anaphase cells, as a proxy for the orientation of cell division at E10.5. This revealed a uniform distribution of cell division orientation in both dorsal and ventral regions of the neural tube (Fig. 5F). Together these results suggest that a difference in cell rearrangements, rather than oriented cell division, account for the reduction of anisotropic tissue growth in the pMN domain.

In summary, the experimental observations are consistent with a model in which tissue growth is resisted by forces which are larger in the AP direction, causing anisotropic growth of the tissue. This effect is lessened in slow-growing epithelia. Thus, clones in the rapidly growing pD domain become more anisotropic after a fixed period of time than clones in the more slowly growing pMN domain. When the tissue grows anisotropically, it does so by biasing the direction of T1 transitions, rather than by biasing the orientation of cell divisions.

## DISCUSSION

To understand the mechanisms by which tissue pattern, mechanics and growth are coupled in the vertebrate neural tube, we used experimental data to construct a mechanical model of the developing neuroepithelium. This allowed us to explore how proliferation and differentiation of individual cells, together with global mechanical constraints, influence the spatiotemporal dynamics of pattern formation in the tissue. Previous observations indicated that there are differences in the anisotropy of the shape of clones in different DV regions of the neural tube (Kicheva et al., 2014), however, how this anisotropy emerged was not understood. Our simulations and analysis indicate that, in the presence of global mechanical constraints, local differences in growth rate create local differences in the anisotropy of tissue growth, consistent with experimental observations. The analysis suggests an explanation for how an isotropic process, such as cell differentiation, can affect an anisotropic process, such as the direction of clonal expansion, given that global mechanical properties (here, dissipation) are anisotropic.

We adopted the well-established vertex model framework to describe neuroepithelium growth (Nagai and Honda, 2001; Farhadifar et al., 2007; Smith et al., 2012; Fletcher et al., 2013). It provides a scalable and computationally efficient means to understand how tissue morphogenesis is influenced by the combined effect of cell shape, forces generated by growing cells and external mechanical constraints. However, one of the challenges of modelling the neural tube epithelium resides in the 3D dynamics of neural progenitors. Similar to many pseudostratified epithelia, cells within the neuroepithelium undergo IKNM (Sauer, 1935) in which cell nuclei migrate between the apical and basal surfaces in synchrony with the cell cycle. Previous approaches have introduced a time dependence to the target area (Canela-Xandri et al., 2011; Kursawe et al., 2018) and we extended the formalism by including the effect of the IKNM and cell cycle on the preferred target apical area. This allowed us to determine mechanical parameters from experimental images of the apical plane, without requiring 3D reconstruction of the neural tube. We could thus recapitulate properties of the pseudostratified dynamics of the tissue without compromising the computational efficiency of the model.

To identify the mechanical parameters of the model, we varied the cell tension, contractility and dissipative forces and compared descriptors of cell and tissue geometry in the resulting simulations with experimental data. The parameter values that produced the highest correlations with experimental data reside in the region of parameter space in which the unperturbed ground state is represented by hexagonal packing (Nagai and Honda, 2001; Gibson et al., 2006; Farhadifar et al., 2007; Magno et al., 2015) and there is a negative correlation between tension and contractility. This is as expected (Kursawe et al., 2018) and in line with parameters used in previous epithelial vertex models (e.g. Farhadifar et al., 2007). Importantly, a model without IKNM yielded a different set of parameters (Fig. S2A) and a poorer correlation with the experimental data (Fig. S2B).

A notable property of neural tube growth, observed in the brachial region at E11.5 of mouse development, is that most of the tissue extends faster in the DV direction than in the AP direction (Kicheva et al., 2014). We found that cell divisions do not show a preferred orientation in the epithelial plane, hence this anisotropy of tissue growth must arise from mechanical constraints. To model this, we assumed that the overall growth of the tissue was resisted by drag forces with different coefficients in the two directions. In general, the sources of resistive forces in epithelia are poorly understood (Alt et al., 2017). In the neural tube, it is possible that expansion is mechanically constrained by the adjacent tissues. Thus, the laterally located somites might affect radial expansion, whereas the process of axis elongation through the addition of cells to the caudal end of the neural tube (Bénazéraf et al., 2010; Mongera et al., 2018) might contribute to forces acting in the AP direction. Obtaining mechanical measurements, such as tension and pressure, using force inference methods (Chiou et al., 2012) within the mouse neural tube *in situ* might provide insight; however, assaying these properties *in vivo* without surgical disruption is difficult or impossible. Similarly, the inaccessibility to live imaging of the unperturbed apical surface, which forms the internal face of the intact neural tube, remains a challenge that hinders obtaining kinematic data on cell shape and movement. Nevertheless, imposing asymmetric drag forces in the simulations generated predictions concerning cell shape, the orientation of the major axis of cells and cell division orientation that were borne out by the experimental data (Fig. 5).

Although the nature of the resistive forces warrants further investigation, the simulations revealed that the anisotropic growth of the tissue resulted from cell rearrangements, rather than changes in cell shape. Thus, T1 transitions were preferentially oriented such that it was more likely for an edge in the AP direction to be replaced by one in the DV direction (Fig. 5A) and the cell intercalation that resulted from T1 transitions contributed to the tissue extension in the DV direction.

Encouraged by the similarity between simulations and experimental data, we used the model to examine the clonal spread in different progenitor domains. The shape of clones was anisotropic throughout most of the neural tube, but clones in the pMN domain were smaller and rounder (Kicheva et al., 2014). The pMN domain is distinguished by a high rate of progenitor loss due to terminal differentiation (Ericson et al., 1992; Kicheva et al., 2014; Sagner et al., 2018), which causes the smaller clone sizes. However, the basis for the difference in clone shape was unclear. pMN cells are molecularly distinct from other progenitors and one possibility was that cell orientation or arrangement was under local molecular control. For example, pMN progenitors express different sets of adhesion molecules than adjacent domains (Rousso et al., 2012), raising the possibility that cell-cell communication plays a role in shaping the pMN domain. Strikingly, however, the model showed that the experimentally observed anisotropy in tissue growth could be reproduced simply by the increased differentiation rate of pMN progenitors in conjunction with the global difference in the resistive forces in AP and DV directions. The difference in resistance causes the tissue to become increasingly more anisotropic with time. Furthermore, increasing the differentiation rate or decreasing the proliferation rate causes the tissue to grow more slowly and become less anisotropic over a given period of time. Hence, the net growth rate is a governing factor that influences the degree of anisotropy, with slow growth being more isotropic.

We found that this change in tissue shape anisotropy over time not only depends on the overall growth rate of the tissue, but also on the relative magnitudes of the proliferation and differentiation rates. Increased differentiation, which removes cells from the epithelium, facilitates cell rearrangements and effectively increases the fluidisation of the tissue (Ranft et al., 2010). This alters the degree of anisotropy that is achieved for a given tissue size compared with growth without differentiation. In summary, the difference in the growth regime between domains influences the degree of tissue anisotropy. Further theoretical investigation will be needed to understand the exact relationships between growth anisotropy, the rate of proliferation and differentiation.

Our analysis shows that the orientation of cell divisions in experimentally observed tissues, as well as in simulations, is random. This suggests that cell division orientation does not contribute to anisotropic tissue growth (Bittig et al., 2008; Li et al., 2014). A surprising observation in our data is that, despite preferential cell elongation in the DV direction, cell division orientation is apparently random in the AP/DV plane; this could be explained by the decrease in elongation observed in mitotic cells (Fig. S8). Cell division has been found to be frequently oriented along the longest planar axis of a cell (Baena-López et al., 2005; Mao et al., 2011; Wyatt et al., 2015; Seldin and Macara, 2017 preprint), although this is not always the case and external and internal cues can determine the orientation of cell division in a shape-independent manner (Gong et al., 2004; Bowman et al., 2006; Konno et al., 2008; Gillies and Cabernard, 2011; Bosveld et al., 2016; Finegan et al., 2019). In the spinal cord, the plane of cell division is regulated along the apicobasal axis and is accompanied by rotations of the metaphase plate (Morin et al., 2007). This regulation is important for maintaining the integrity of the epithelium. Our data are consistent with the idea that the apicobasal orientation of the spindle is the dominant mode of regulation in the spinal cord, with no specific mechanism acting to orient the planar angle. It could be that the random orientation of divisions in the epithelial plane and the decoupling from cell shape is necessary to achieve efficient apicobasal orientation (Morin et al., 2007). Further studies will be necessary to investigate this thoroughly.

A consequence of the difference in clone shape between pMNs and other progenitor subtypes is that cells in all progenitor domains expand at equal rates along the AP axis, despite the overall smaller size of pMN clones. Thus, there is no net AP movement between progenitor domains and cells stay in register as development proceeds. This means that cells in different DV domains with the same AP identity remain adjoining. As AP identity is established early during neural development, maintaining position relative to other cells in the epithelium may be important for the later assembly of position-appropriate functional neuronal circuits.

In conclusion, we described a vertex model of a pseudostratified epithelium and used it to study the growth of the neural tube and how cell differentiation influences clone shape. In future work, we wish to couple this tissue model to quantitative descriptions of the spread of morphogens that pattern the tissue and the gene regulatory networks that specify neuronal subtype identity. In this way, we hope to gain insight into the coupling of growth and patterning in the neural tube and understand how the position, precision and proportions of cell types are achieved.

## MATERIALS AND METHODS

### Experimental data analysis

All animal procedures were performed in accordance with the Animal (Scientific Procedures) Act 1986 under the UK Home Office project licences PPL80/2528 and PD415DD17. E10.5 and E11.5 mouse embryos were collected and processed for dissection, fixation, immunostaining and flat-mounting as previously described (Kicheva et al., 2014). Primary antibodies used were: mouse anti-ZO-1 (33-9100, Zymed Laboratories, 1:100), rabbit anti-phospho Histone H3 (06-570, Millipore, 1:1000), mouse anti-Aurora B (AIM1, BD Transduction Laboratories, 1:500), rabbit anti-Olig2 (AB9610, Millipore, 1:1000) and mouse anti-Pax3 (AB_528426, DSHB, 1:20). For the experimental clone shapes, we re-analysed the data in Kicheva et al. (2014). Cell elongation was calculated from the second moment matrix of the polygon representing the cell by taking the square root of the ratio of the largest to the smallest eigenvalue. Cell angle orientation was defined by *arctan*(*x*1/*y*1), where *x*1, *y*1 represent the AP and DV component of the eigenvector corresponding to the largest eigenvalue.

To quantify the cell division angle, E10.5 embryos were stained with DAPI, phospho-H3 and Aurora B to mark dividing cells, and Pax3 and Olig2 to mark the dorsal and pMN domains, respectively. Spindle rotation ceases in anaphase (Morin et al., 2007). Cells in this phase were identified by low levels of phospho-H3, separated sister chromatids and Aurora B staining associated with the central spindle and only these cells were considered for the analysis (Fig. 5B, bottom). The orientation of the chromosomes with respect to the AP axis of the embryo was measured.

For the analysis of cell geometries, we used images of flat-mounted embryos of approximate size 80×80 μm. Images taken within the dorsal half are composed of Pax7+ pD progenitors, whereas images taken in the ventral part of the neural tube contained up to 50% pMN cells with the remainder being progenitors of the p2-p0 domains. Images were processed using the Fiji plug-in ‘Packing Analyzer v2.0’ (Aigouy et al., 2010), which segments the image, classifies cell edges and vertices, and measures cell areas, perimeter and neighbours. Segmentation mistakes were manually corrected.

### Vertex model description

Cells are represented as polygons with straight edges connecting vertices. Cells are enumerated by *α*=1, …, *N*_*c*_ and vertices are enumerated by *i*=1, …, *N*_*v*_. The evolution of each cell in these models is governed by the motion of its vertices, which are typically assumed to obey deterministic equations of motion. It is usual to make the simplifying assumption that the motion of vertices are overdamped (Drasdo, 2000), and inertial terms are small compared with dissipative terms. This leads to first-order dynamics. The evolution of the position *r*_*i*_ of vertex *i* is determined by:(4)
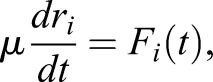
where *F*_*i*_(*t*) denotes the total force (except drag) acting on vertex *i* at time *t* and *μ* denotes its drag coefficient. The main difference between models lies in the definition of the force *F*_*i*_ that can be derived from an energy function, *E*, which includes the different cell-cell interactions. In our model we use a modification of the energy function described in Farhadifar et al. (2007), by including time dependence of the target area term, *A*_α_^0^(*t*). Thus:(5)
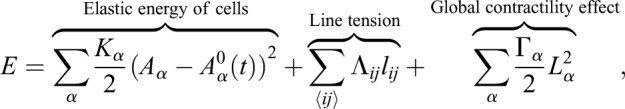
for which 
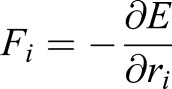
.

The first term describes an area elasticity with elasticity coefficients *K*_*α*_, for which *A*_*α*_ is the area of cell *α* and *A*_α_^0^(*t*) is the preferred area at time *t* (the preferred area will be related to the apicobasal nuclear position in this 2D model, Eqn 1). The second term is dependent on the length of cell-cell junctions, representing adhesion/tension energy. It introduces the energy associated with bonds between each cell and its neighbours, where Λ_*ij*_ is a constant and *l*_*ij*_ denotes the length of the junction linking vertices *i* and *j*. When Λ_*ij*_ is negative, cell boundaries tend to expand; when it is positive, the edges tend to shrink. The sum of 〈*ij*〉 is over all bonds. The third term describes the contractility of the cell perimeter *L*_*α*_ by a positive coefficient Γ_*α*_, when it is small, contractile forces are small compared with those from area elasticity.

We assume all parameters are the same in each cell or edge, so *K*_*α*_=*K*, Λ_*ij*_=Λ, and Γ_*α*_=Γ. In this case, the final term in Eqn 5 can be expressed as a sum over cell perimeters and combined with the final term to give 
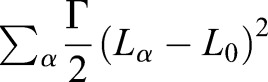
, where the target perimeter *L*_0_ is given by − Λ/2Γ. The added constant term is irrelevant, as the dynamics only depend on changes in energy.

### Vertex model implementation

The model is implemented with a custom Python code (available in Bitbucket: https://bitbucket.org/Pigueco/vertex_model_python_2.7) using the Euler method to solve the equation of movement for each vertex, Eqn 4. We nondimensionalised in time and space by taking 460 s as a unit of time and using an area of 23 μm^2^. The units of force are arbitrary. The tissue is initialized as a hexagonal mesh of 10 by 10 cells, or 15 by 15 cells for simulations with pMN domain. The initial tissue is allowed to evolve for 30 h (biological time) to generate a vertex distribution close to steady state. At this point, the time is reset and the vertex distribution is taken as the starting point for the simulations described in this study.

To accommodate topological transitions in the simulations, we introduced the possibility of T1 transitions. During a T1 transition an edge below 3% of the average edge length of the tissue is eliminated and a new edge of length *l*_*new*_ expands perpendicular to the old edge (values given in Table 2). If the rearrangement results in the formation of a two-sided cell, the cell is removed from the epithelium.

Division occurs when the cell is in M-phase (*t̃* > *t*_*G*1_ + *t_S_* + *t*_*G*2_) and the volume of the cell exceeds a critical value, *A*_*c*_. For a cell to divide, a new edge is introduced by creating two new vertices. The location of the first vertex is chosen as the midpoint of a randomly selected edge of the dividing cell with probability proportional to the edge length. The other vertex is the midpoint of the opposite edge; if the cell has an odd number of sides the second edge is the closer mid edge. The newly generated sister cells then commence the next cell cycle.

In order to define the frequency of cell divisions in the model, we defined the proliferation rate *λ* as *d*/(*Ñ*Δ*t*), where *d* is the number of division events in a small time interval Δ*t*, and *Ñ* is the average number of cells in the tissue during Δ*t*. For a proliferating tissue in which differentiation does not occur, this estimate of *λ* is equivalent to the effective rate of tissue growth *k*=ln(*N*_*C*_(Δ*t*)/*N*_*C*_)/Δ*t*, where Δ*t* is a time interval, *N*_*C*_ is the number of cells in the tissue at the start of the interval and *N*_*C*_(Δ*t*) the number of cells at the end of the interval. To match the experimental data (Kicheva et al., 2014; Table 1), in the simulations we aimed to obtain a proliferation rate of 0.05 h^−1^. For a proliferating tissue without differentiation *λ*=ln(2)/*t*_*T*_ where *t*_*T*_ is the total cell cycle time. Thus, *λ*=0.05 h^−1^ corresponds to an average cell cycle length of 13 h, which is 10^5^ simulation time steps (Tables 1 and 2). In Fig. S6 *t_T_*=13 h is used for the condition with fixed proliferation and varied differentiation. In tissues with high levels of differentiation, however, the proliferation rate is an effective rate because at any one time a fraction of cells present in the tissue will not further divide. Thus, in a tissue with a differentiation rate of 0.1 h^−1^ and effective proliferation rate of 0.05 h^−1^ the cell cycle time of the dividing cells is on average shorter and corresponds to 11.7 h. Note that the estimates of cell cycle duration given in Table 1 are based on fractions of dividing cells from fixed images and are therefore effective measurements. The values of the proliferation rates given in Fig. 4F and Fig. S6 are *ln*(2)/*t*_*T*_, where *t*_*T*_ is used in simulations to determine the evolution of the target area and the minimum time at which cells can divide. For proliferation rate 0 h^−1^, *t*_*T*_ is set to 13,000 h. Number of neighbours, cell area and cell perimeter distributions in simulations were compared with output using code from Smith et al. (2012).

### Growth of the tissue

We model the neural tube as a torus with two radii, *R* and *H*. The torus can grow in both radial directions. The growth in *R* is resisted by a drag force of magnitude 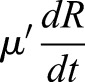 per cell. The forces are balanced so drag forces are of the same total magnitude, as the other forces.

Thus, its growth is determined by a balance between the potential forces and drag:(6)
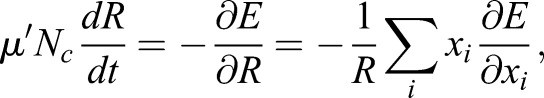
where *x*_*i*_=*Rθ*_*i*_ is the coordinate that we use for the *i*th vertex in the dorsoventral direction (Supplementary Materials and Methods, section I).

Equivalently, we calculate growth in the perpendicular direction, *H*, using the drag coefficient, 2*μ*″.

The tissue aspect ratio (AP/DV) was measured using the whole-tissue AP length and DV length. The AP/DV clone aspect ratio was measured as defined in Kicheva et al. (2014) to enable comparison between experimental data and simulations. In computing the ratio, one nondimensional unit of length is subtracted from the mean AP and mean DV lengths of clones.

### Statistical analysis

Statistical analysis details are documented in figure legends and captions. To compare the distributions of cell area, perimeter and number of neighbours of the experimental and simulated data in Fig. 3A and Fig. S2A we quantified the Kolmogorov–Smirnov (K–S) statistic, which measures the difference between distributions. We used mean distributions for the comparison, derived by averaging across 11 images at E11.5 for the experimental data, and 10 runs of the model for the simulated data. We then report the K–S statistic, which represents the largest difference between the mean distributions. Other quantities compared between simulations in Fig. 3A,B and Fig. S3A – cell area standard deviation, cell perimeter standard deviation, DV length of tissue and AP/DV ratio of tissue – were absolute values of the differences between the experimental values and simulation values. The difference between the mean value of 11 images and each of 10 simulation runs was taken and then the mean of these 10 values. In Fig. 3B and Fig. S3A, the values for AP/DV ratio and DV length were then summed.

## Supplementary Material

Supplementary information
