# Supplementary material for: Neuronal differentiation influences progenitor arrangement in the vertebrate neuroepithelium
Source: Development. 2019 Dec 4;146(23):dev176297. doi: 10.1242/dev.176297 (PMC6918779; doi:10.1242/dev.176297)
Supplement: Supplementary information [file develop-146-176297-s1.pdf]

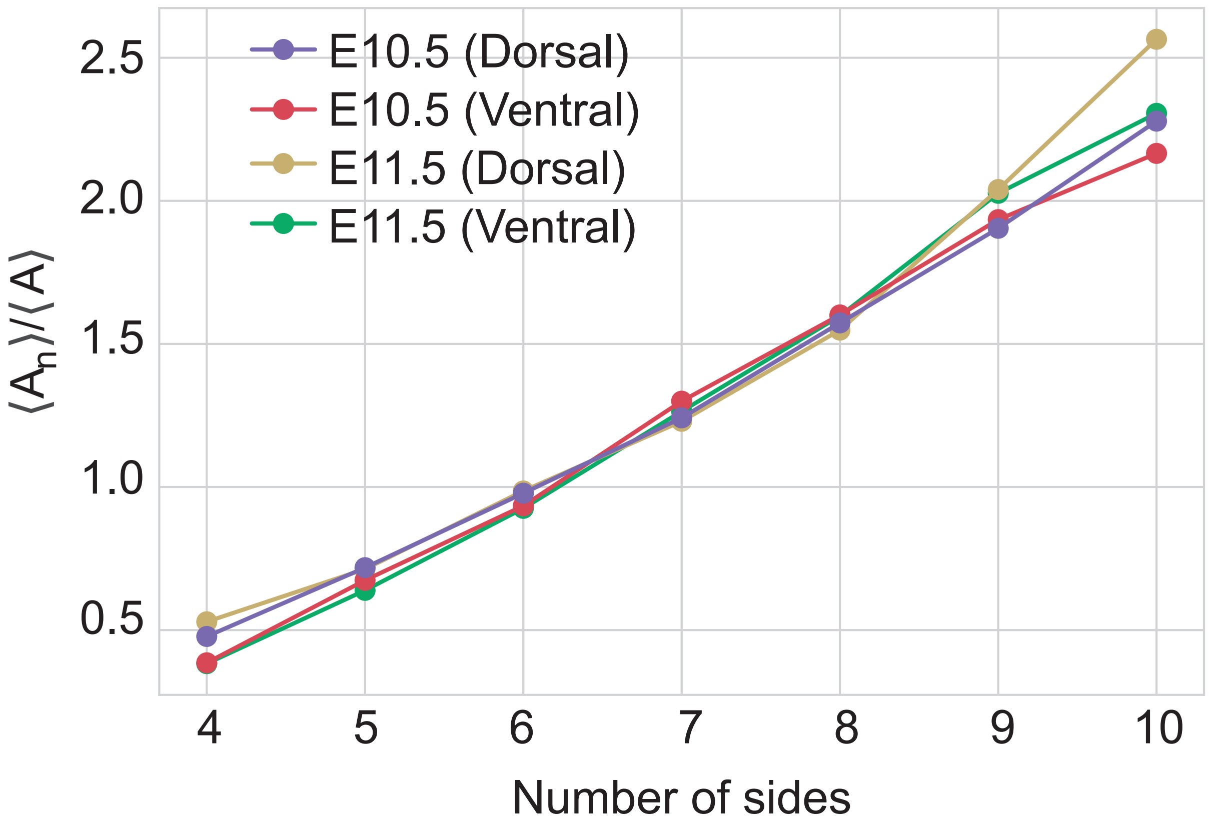

Fig. S1. Average area of different polygon classes Mean areas of different polygon classes normalised to the average area of cells, $\langle A_n \rangle / \langle A \rangle$, in experimental data from the ventral and dorsal domains at mouse neural tube stage E10.5 and E11.5.

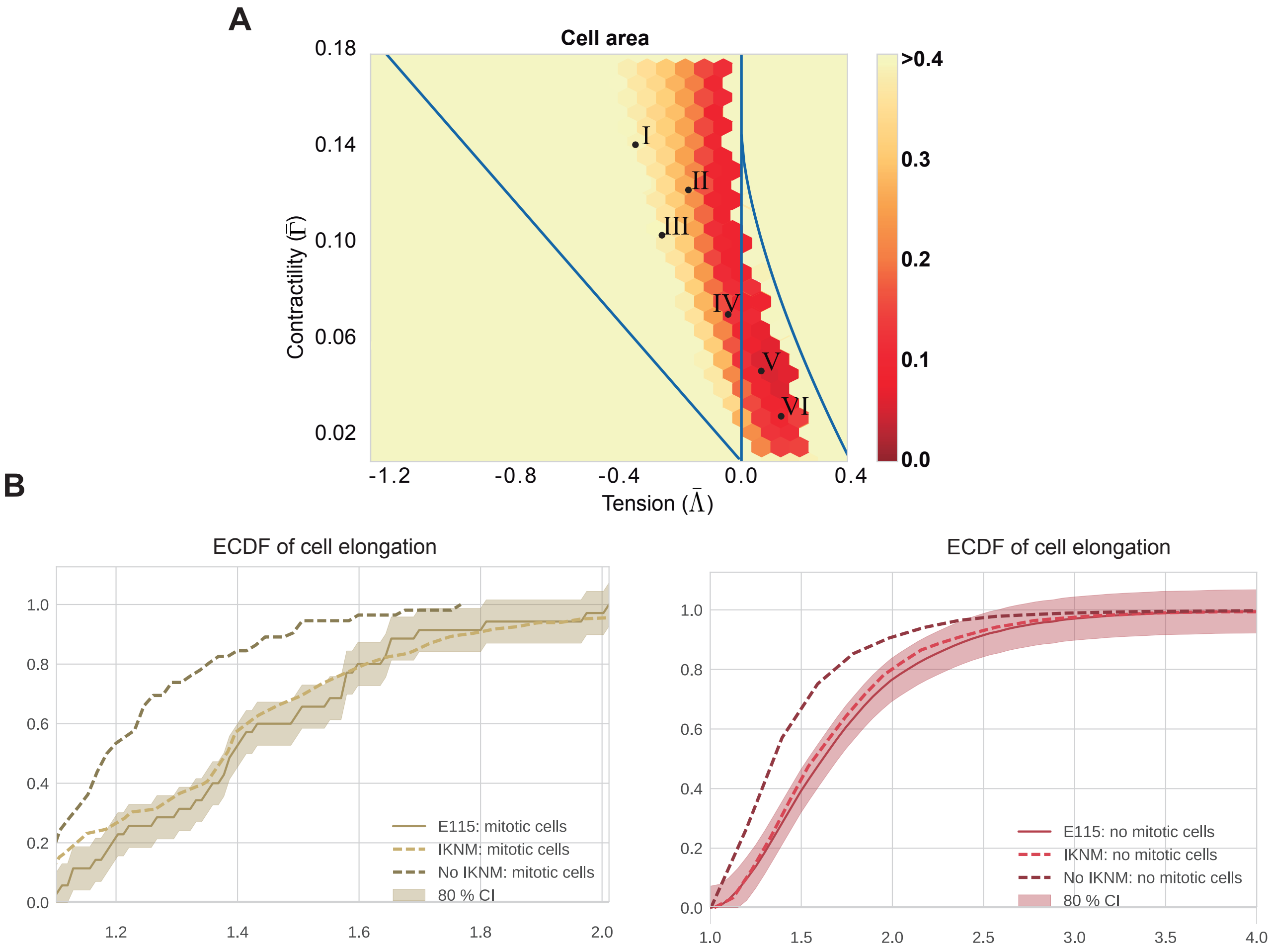

Fig. S2. Simulations without IKNM match experimental data less well A) Map of the average of maximum distance between the experimental and simulated empirical cumulative distribution function of cell area distribution for a model without IKNM (constant target area). Experimental data corresponds to E11.5 embryos and simulations correspond to 10 simulations per point in the parameter space. Roman numeral marked points indicate the mechanical parameters selected, ($\bar{\Lambda}$, $\bar{\Gamma}$) in Fig. 3A. B) Empirical cumulative distribution function (ECDF) of cell elongation in simulations with and without IKNM using mechanical parameter V in Fig. 3A compared to E11.5 experimental data of mitotic and non-mitotic cells (left and right respectively).

Development • Supplementary information

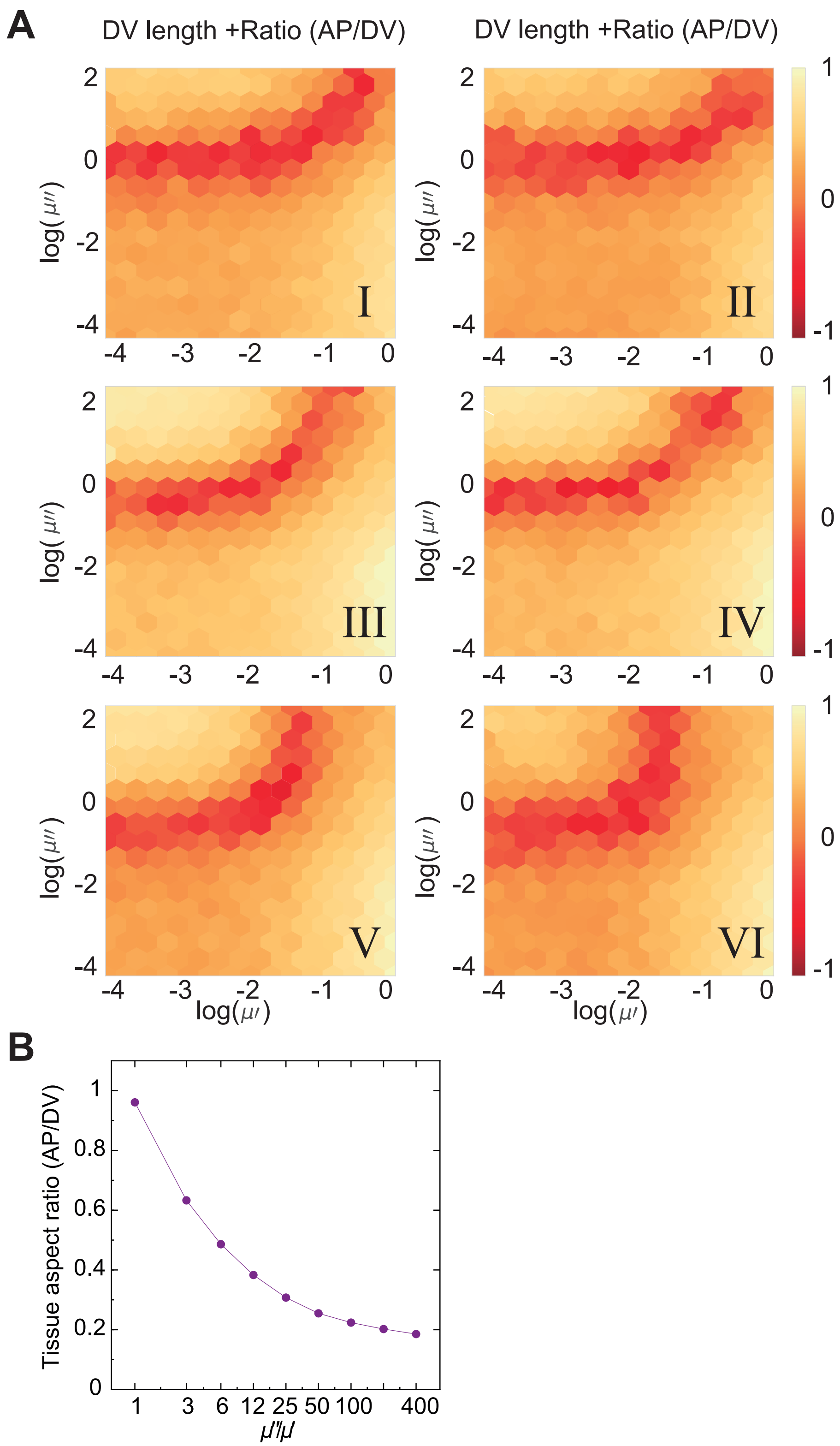

Fig. S3. Determining drag coefficients that generate anisotropic growth in simulations A) Heatmap indicating the difference between simulation and experimental tissues for different values of the drag coefficients μ' and μ''. The absolute value of the difference in the change in DV length of the tissue over 48h plus the absolute value of the difference in the final tissue aspect ratio (AP/DV) between simulations and experimental data was taken and then this quantity was averaged over 10 simulations. Colour map shows the average difference (log scale) from 10 different simulations per point with the experimental data from E11.5 embryos. Other parameters are indicated in Table 2. Roman numerals indicate mechanical parameters used $(\bar{\Lambda}, \bar{\Gamma})$ in Fig. 3A. B) The AP/DV aspect ratio of simulated tissues at the end of the simulation (72h) for a range of μ''/μ' ratios. $\mu'' = 1$ is held constant and μ' is varied from 0.0025 to 1. The proliferation rate is $0.05h^{-1}$ and there is no differentiation. Parameter regime V is used. The reported errors are SE.

Development • Supplementary information

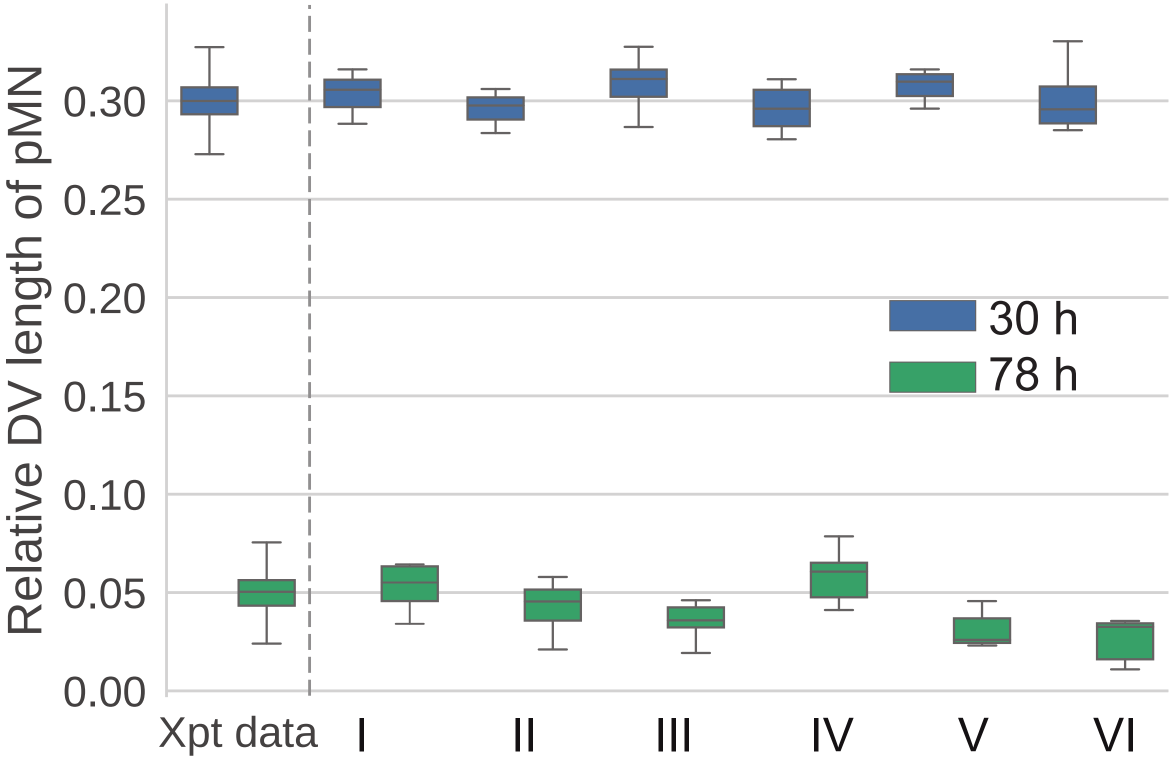

Fig. S4. The effect of introducing differentiation on DV length of the pMN domain DV length of the pMN domain relative to the total DV length of the tissue at the point pMN is introduced to simulations (blue) and after 48 hours (green). Differentiation rate in pMN domain $0.1h^{-1}$. Other parameters are indicated in Table 2. These time points correspond to experimental samples (Xpt data) from E9.5 and E11.5 mouse embryos, respectively. The DV length at these developmental stages was measured in (Kicheva et al. (2014)).

Development • Supplementary information

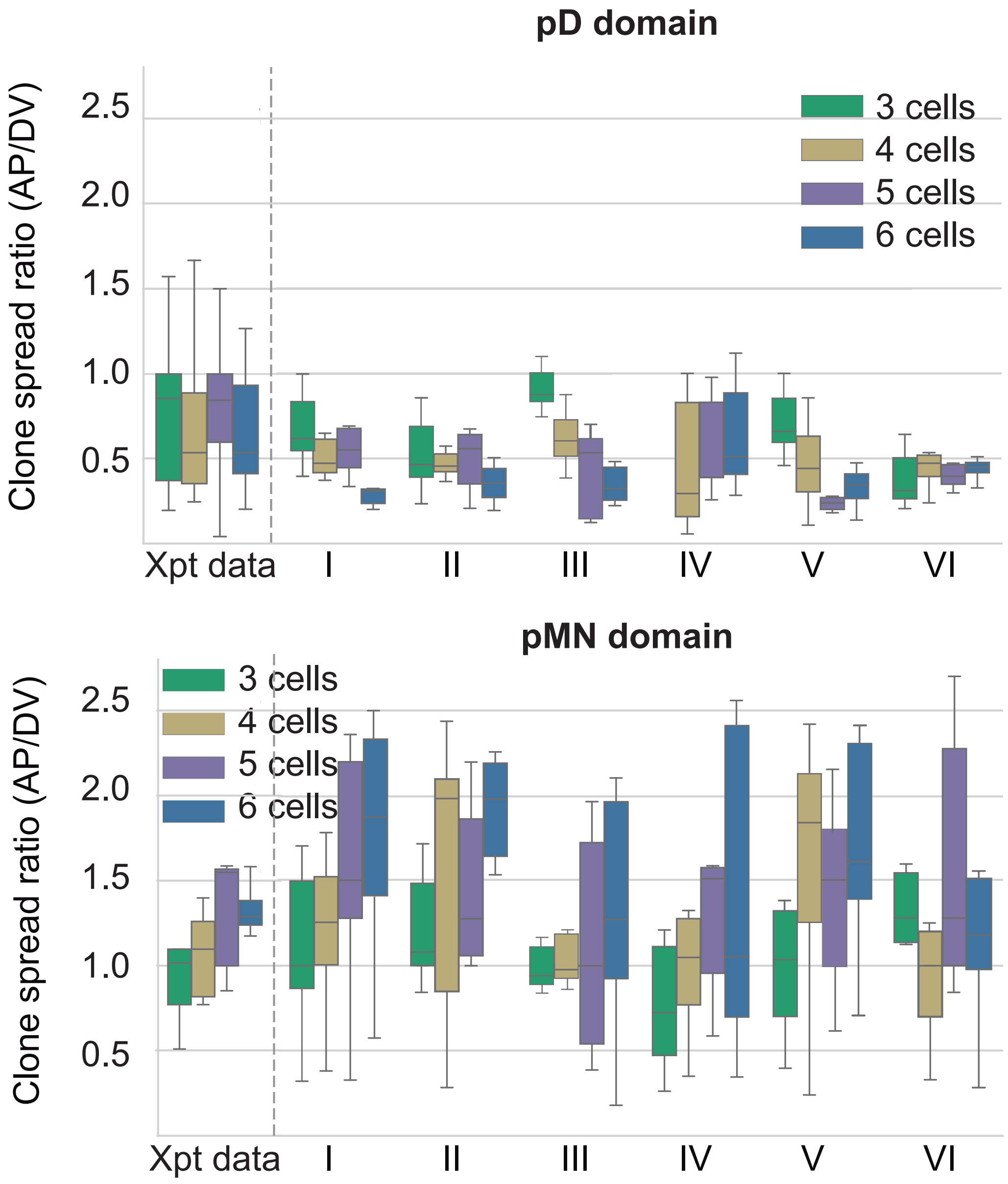

Fig. S5. Shape of clones of different size in the pD and pMN domain The AP/DV aspect ratio for clones containing the indicated number of cells per clone (3 green, 4 yellow, 5 purple, 6 blue) in the pD and pMN domains. Experimental samples (Xpt data) are compared to the result of 12 simulations per mechanical parameter set $(\bar{\Lambda}, \bar{\Gamma})$ from Fig. 3A. Differentiation rate in pMN is $0.1h^{-1}$. Other parameters are indicated in Table 2.

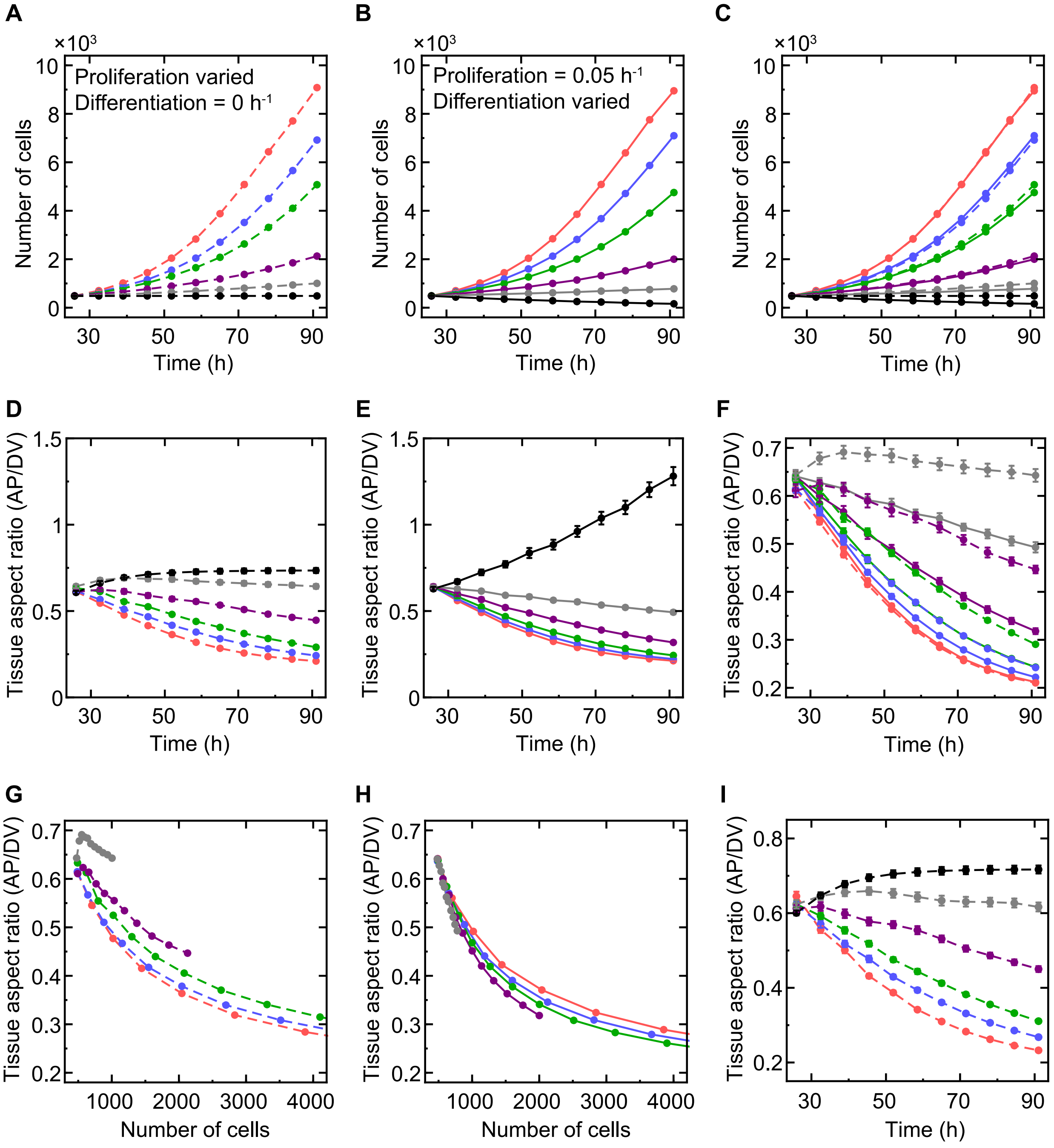

Fig. S6. Effect of proliferation and differentiation rate on the size and shape of simulated neuroepithelial tissue A) Number of cells in simulated tissues as a function of time for different proliferation rates and no differentiation. The proliferation rates from top to bottom: $0.05h^{-1}$ (red), $0.04h^{-1}$ (blue), $0.03h^{-1}$ (green), $0.02h^{-1}$ (purple), $0.01h^{-1}$ (grey), $0h^{-1}$ (black). In A-I: the simulation starts with an initial period of ($t < 26h$) during which the proliferation rate is $0.05h^{-1}$ and there is no differentiation. Afterwards proliferation or differentiation is altered as indicated. In A-H parameter regime V is used. B) The number of cells as a function of time for different differentiation rates and fixed proliferation rate = $0.05h^{-1}$. The differentiation rate is $0\ h^{-1}$ (red), $0.01h^{-1}$ (blue), $0.02h^{-1}$ (green), $0.035h^{-1}$ (purple), $0.05h^{-1}$ (grey), $0.075h^{-1}$ (black). The parameters were chosen so that the net growth rate in B is similar to that in A for curves of the same colour (except black). C) Overlaid data from A and B. D) Aspect ratio of the tissue AP to DV extension for varied proliferation and no differentiation (same as main Fig. 4F, parameters as in A). E) As D but for varied differentiation and fixed proliferation (parameters as in B). F) Overlaid data from D and E. Only simulations with positive net growth rate are plotted (no black trajectories). G, H) The tissue aspect ratio (AP/DV) from D and E as a function of cell number. Only conditions with positive growth rate are shown. I) The tissue aspect ratio (AP/DV) for varied proliferation and no differentiation in parameter regime VI. The proliferation rates and colour convention as in (A). Data A-I was averaged over 12 independent simulations for each condition. The reported errors are SEM. If error bars are not visible the SEM was very small (<2%).

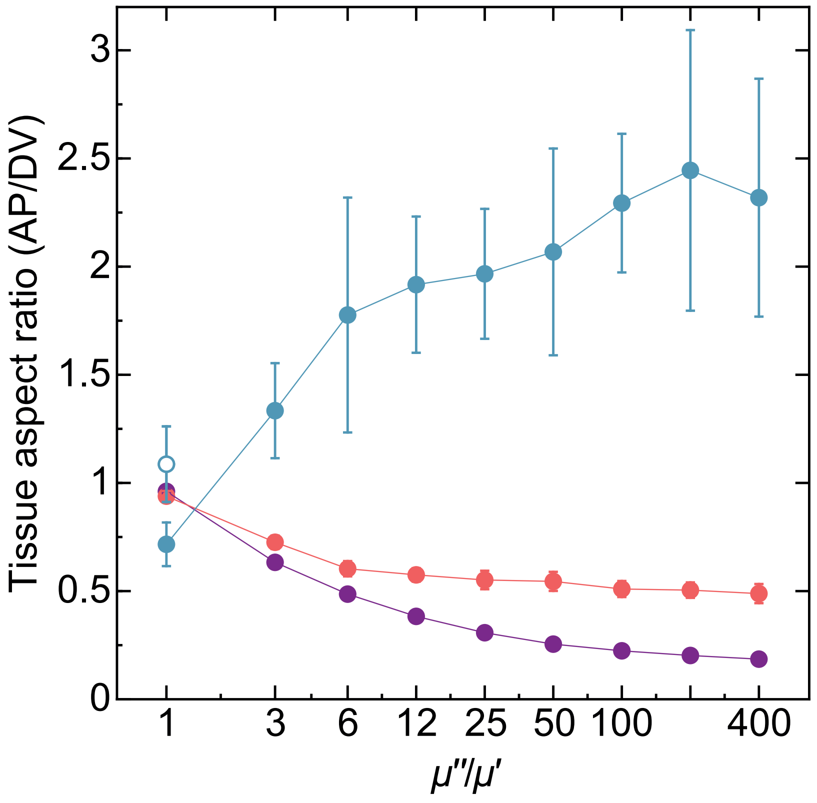

Fig. S7. Influence of the ratio of the AP/DV drag coefficients on the shape of tissue with different rates of differentiation Increasing the differentiation rate increases the AP/DV aspect ratio of simulated tissues for all μ''/μ' ratios larger than 1. The AP/DV aspect ratio at the end of the simulation (72h) is reported. The simulation starts with an initial period of ($t < 26h$) during which the proliferation rate is $0.05h^{-1}$ and there is no differentiation. Afterwards differentiation is altered: $0h^{-1}$ (purple, same as in Fig. S.3B), $0.05h^{-1}$ (red), $0.1h^{-1}$ (solid light blue). The indicated μ''/μ' ratio is fixed from the start. The parameter regime V is used. In the case μ''/μ'=1, the final AP/DV aspect ratio is <1. This is because the tissue is initialized with hexagonal mesh of 10 by 10 cells (honeycomb pattern) that has the AP/DV ratio = $\sqrt{3}/2 \approx 0.87$. If the tissue is initialized with hexagonal mesh of 10 cells in DV direction and 12 cells in AP direction (initial AP/DV ratio ≈ 1.04), then the end result is also isotropic as expected (empty light blue dot). The reported errors are SE.

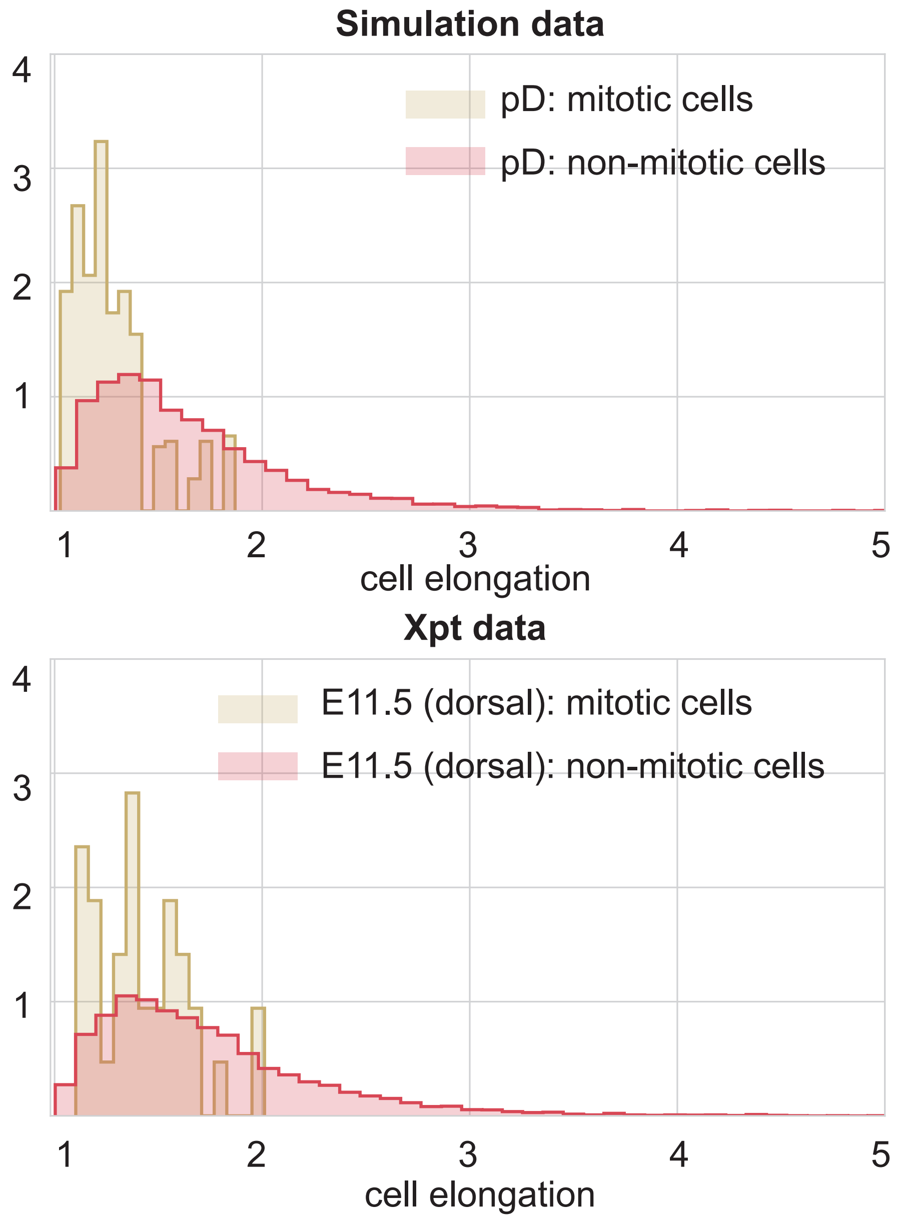

Fig. S8. Cell elongation in simulated tissue and experimental data Histogram of cell elongation in cells close to mitosis (brown) and non-mitotic (red) cells in simulations and experimental data in the dorsal domain. In the experimental data, the 2.5% of cells with largest area were considered mitotic. For the simulations, parameter regime V was used.

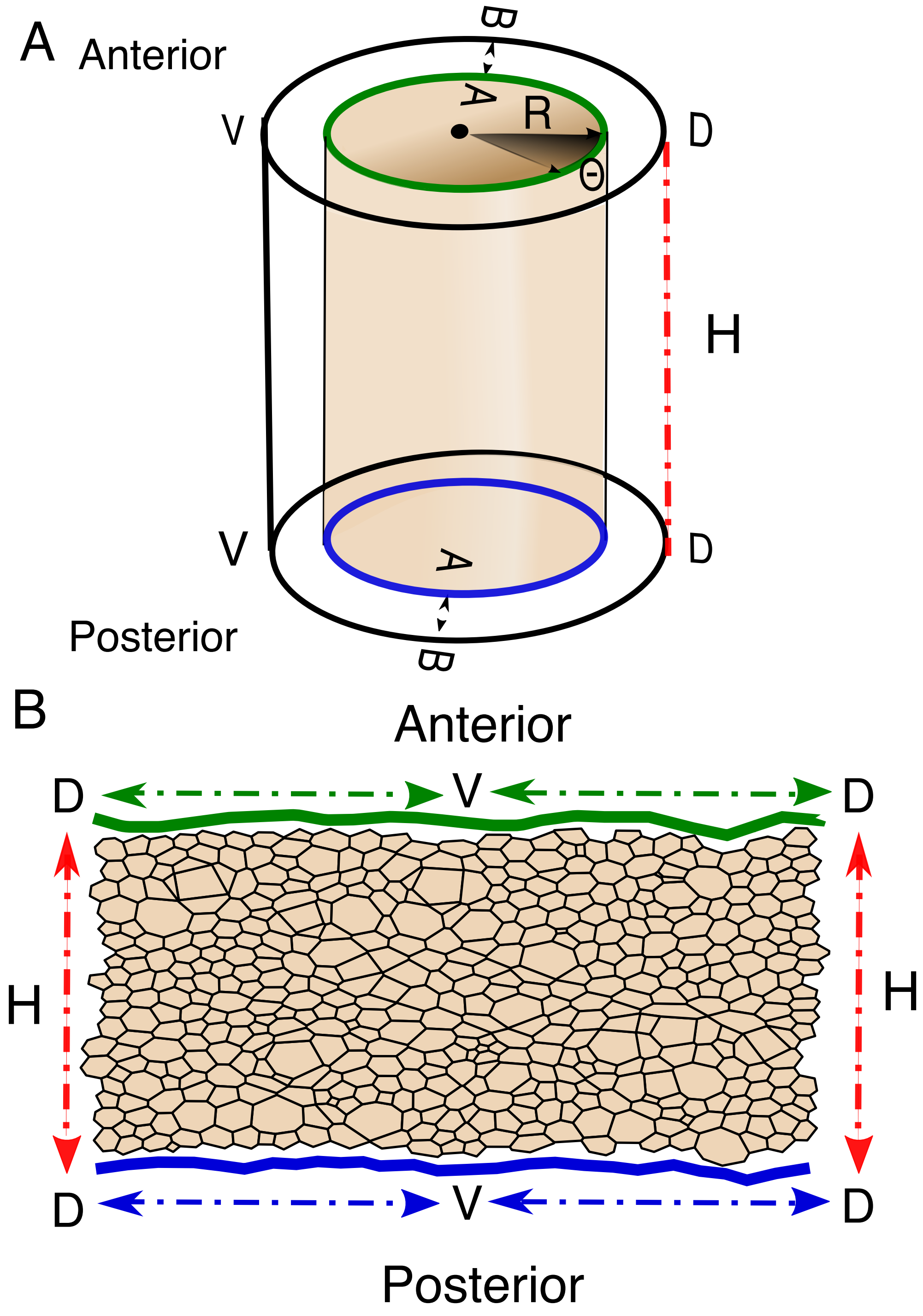

Fig. S9. Cylindrical representation of the neural tube A) Cylindrical representation where R is the radius, θ is the angle and H is height of the cylinder and the z-axis is along this length. B) Polygon representation of the apical junctional network.

Development • Supplementary information

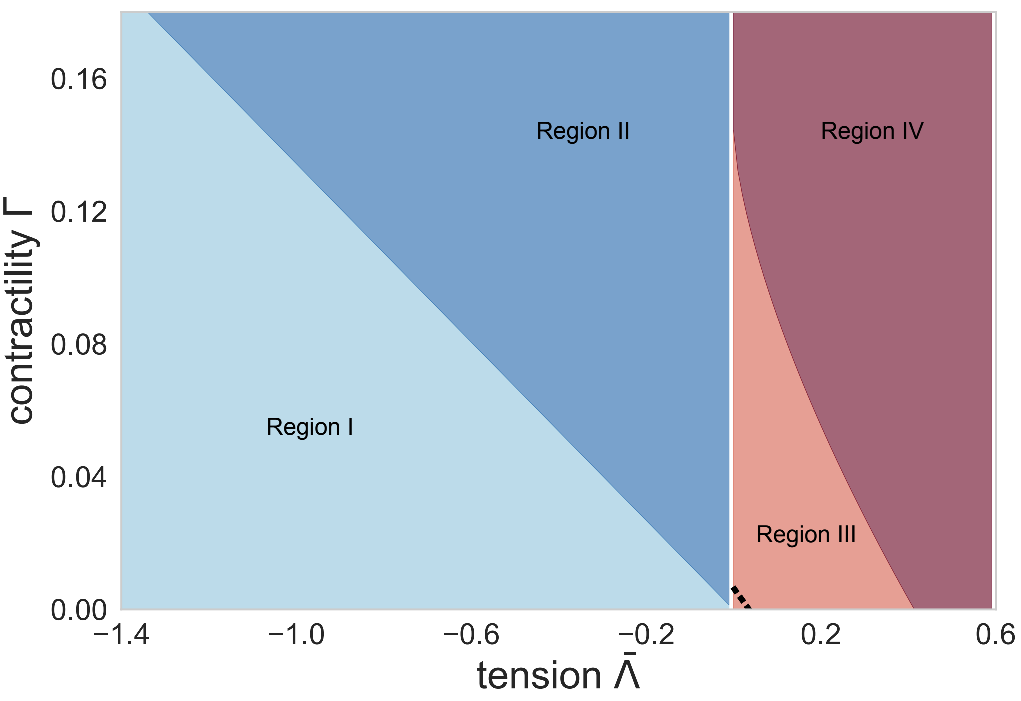

Fig. S10. Phase diagram Phase diagram for the biophysical parameter space for hexagonal cells in terms of $\bar{\Lambda}$ and $\bar{\Gamma}$.

Supplementary Materials and Methods: Neuronal differentiation influences progenitor arrangement in the vertebrate neuroepithelium

I. CYLINDRICAL REPRESENTATION

The domain of the vertex model is a cylinder, the radius, R, and height, H, of which can change in time. A natural set of coordinates for the vertex i within the cylinder are $(\theta_i, \tilde{z}_i)$, where θ_i are cylindrical polar angles and $\tilde{z}_i = z_i/H$, where z_i is the coordinate in the direction along the length of the cylinder, see Fig. S9. Note that in simulations we actually impose periodic boundary conditions also in the direction along the length of the cylinder. The movement of vertices is determined by an energy function, E.

We assume overdamped movement, with the drag coefficient for vertices within the cylinder (whilst the cylinder size is fixed) being μ. Therefore:

$$\mu R \frac{d\theta_i}{dt} = -\frac{1}{R}\frac{\partial E}{\partial \theta_i}, \qquad \text{(SM.1)}$$

$$\mu H \frac{d\tilde{z}_i}{dt} = -\frac{1}{H}\frac{\partial E}{\partial \tilde{z}_i}. \qquad \text{(SM.2)}$$

In addition the cylinder itself can grow. We assume that cells (whose average areas are constant) experience some drag proportional to their speed, both radial and tangential (frictional) as the outside surface of the cylinder expands. Radially the speed of each cell is $\frac{dR}{dt}$ and along the length of the cylinder, the velocities are on average $\frac{1}{2}\frac{dH}{dt}$ relative to a fixed point at one end of the cylinder [1]. Let us define the relevant drag coefficients to be μ' radially and $2\mu''$ along the length of the cylinder. The equation for the radius and the height are determined by the balance between the radial component (the height component) of the potential forces and the accumulated drag on all of the cells:

$$\mu' N_c \frac{dR}{dt} = -\frac{\partial E}{\partial R}, \mu'' N_c \frac{dH}{dt} = -\frac{\partial E}{\partial H}, \qquad \text{(SM.3)}$$

where N_c is the number of cells. We note that the three drag coefficients μ, μ' and $2\mu''$ are likely to be different. We also note when a cell is added its energy is added to E.

The coordinates that we actually use in the vertex model code are given by (x_i, z_i), where $x_i = R\theta_i$ and $z_i = H\tilde{z}_i$, which are effectively rectangular coordinates in a folded out cylinder. The equation for the radius, thus becomes:

$$N_c\mu' \frac{dR}{dt} = -\sum_i \frac{\partial E}{\partial x_i}\frac{\partial x_i}{\partial R} = -\frac{1}{R}\sum_i x_i \frac{\partial E}{\partial x_i}. \qquad \text{(SM.4)}$$

[1] The magnitude of the overall force is the same if we consider the middle of the cylinder as being stationary.

Therefore

$$\frac{dx_i}{dt} = -\frac{1}{\mu}\frac{\partial E}{\partial x_i} - \frac{1}{\mu'}\frac{x_i}{N_c R^2}\sum_j x_j \frac{\partial E}{\partial x_j}. \qquad \text{(SM.5)}$$

Similarly,

$$\frac{dz_i}{dt} = -\frac{1}{\mu}\frac{\partial E}{\partial z_i} - \frac{1}{\mu''}\frac{z_i}{N_c H^2}\sum_j z_j \frac{\partial E}{\partial z_j}. \qquad \text{(SM.6)}$$

In the code, we update x_i and z_i first according to equation (SM.1) and (SM.2), (i.e. in time step Δt we add $R\Delta\theta_i$ and $H\Delta\tilde{z}_i$), then we multiply by a factor $\frac{R+\Delta R}{R}$ and $\frac{H+\Delta H}{H}$ respectively, where we call $\frac{\Delta R}{R}$ and $\frac{\Delta H}{H}$ "expansion" and these are given by $-\frac{\Delta t}{N_c\mu' R^2}\sum_j x_j \frac{\partial E}{\partial x_j}$ and $-\frac{\Delta t}{N_c\mu'' H^2}\sum_j z_j \frac{\partial E}{\partial z_j}$.

II. BIOPHYSICAL PARAMETER SPACE

We study the effect of the biophysical parameters on the cell dynamics. The energy (Eq. 4) is determined by the vertex positions and parameters K, Λ, and Γ. Magno et al. [2015] find parameter regions that yield different behaviours depending on the mechanical parameters.

The energy E for an individual cell is given by:

$$E = \frac{K}{2}(A - A^0)^2 + \frac{\Lambda}{2}L + \frac{\Gamma}{2}L^2. \qquad \text{(SM.7)}$$

The interfacial tension γ and pressure Π are given by

$$\gamma = \frac{\partial E}{\partial L} = \frac{\Lambda}{2} + \Gamma L, \qquad \text{(SM.8)}$$

$$\Pi = -\frac{\partial E}{\partial A} = -K(A - A^0). \qquad \text{(SM.9)}$$

A reduction in cell perimeter reduces the energy and is therefore favoured. This favours cells that take a regular shape, hence we require the interfacial tension to be positive,

$$\gamma = \frac{\Lambda}{2} + \Gamma L > 0. \qquad \text{(SM.10)}$$

This should be true for the equilibrium cell size.

Suppose a cell has a fixed shape and denote the square root of its area by l and its shape index by $s = L/\sqrt{A}$ where L and A are the perimeter and the area cell. The equilibrium size is determined by setting $\frac{\partial E}{\partial l} = 0$:

$$\frac{\partial E}{\partial l} = \gamma\frac{dL}{dl} - \Pi\frac{dA}{dl} = 0, \qquad \text{(SM.11)}$$

thus at equilibrium $\gamma s = 2\Pi l$, and therefore

$$2Kl^3 = -\frac{\Lambda}{2}s + (2KA^0 - \Gamma s^2)l. \qquad \text{(SM.12)}$$

Development • Supplementary information

We note that when $l = 0$, $\frac{\partial E}{\partial l} = \Lambda s$, so the state $l = 0$ is stable if and only if $\Lambda > 0$. Eq. SM.12 has a single positive solution corresponding to an energy minimum if $\Lambda < 0$ (meaning cells converge to a single equilibrium area). If $\Lambda > 0$, Eq. SM.12 can either have zero positive solutions (in which case all cells collapse to zero area) or two positive solutions. In the latter case, the larger positive solution will correspond to an energy minimum and the small positive solution to an energy maximum. Thus cells with area greater than the unstable value will expand their area towards a fixed positive value, while the area of small cells will collapse to zero.

We note that the condition for the state $l = 0$ to be unstable is independent of the target area. In our model, the target area varies slowly compared to the movement of vertices and thus we expect this property to hold for the same parameter values in the model with varying target area too. The condition for regularly shaped cells does depend on the target area, since when interfacial tension is zero, the minimum energy will be attained when cell area is equal to target area. With other parameters fixed, as the target area increases, the interfacial tension will also increase and so cells will tend to be more regular. Thus it is possible that small cells will be irregularly shaped, but larger ones will be regularly shaped. This is because for small cells the adhesive force will dominate the force from the actomyosin ring, whereas for larger cells, the opposite will be true.

The final condition that we need to determine is the condition for there to be two positive steady states instead of zero when $\Lambda > 0$. There are two positive states if and only if

$$A^0 > \frac{\Gamma s^2}{2K} + \frac{3}{4}\left(\frac{\Lambda s}{K}\right)^{2/3}. \qquad \text{(SM.13)}$$

This condition depends on our variable target area. What really matters, however, is whether cells are likely to grow at any size that they attain (i.e. it does not matter if we are in the region with two stable areas if the cells are so small when they are born that they shrink to zero). A necessary condition for cells to shrink from their size at birth is that $\frac{\partial E}{\partial l}\big|_{l=\sqrt{A_c/2}} < 0$ with the target area given by its value at the start of the cell cycle. A sufficient condition is that $\frac{\partial E}{\partial l}\big|_{l=\sqrt{A_c/2}} < 0$ with the target area given by its minimal value. Now

$$\left.\frac{\partial E}{\partial l}\right|_{l=\sqrt{A_c/2}} = \frac{\Lambda}{2}s + \Gamma s^2\sqrt{A_c/2} + 2K\sqrt{A_c/2}(A_c/2 - A^0). \qquad \text{(SM.14)}$$

The cells will thus grow if

$$2K\sqrt{A_c/2}(A^0 - A_c/2) > \frac{\Lambda}{2}s + \Gamma s^2\sqrt{A_c/2}. \qquad \text{(SM.15)}$$

Let us use the notation of Farhadifar et al. [2007], with the A^0 that they use to create the nondimensional parameters given by its mean value, $\langle A^0\rangle$ during the cell cycle in our model. The nondimensional parameters are $\bar{\Lambda} = \frac{\Lambda}{K\langle A^0\rangle^{3/2}}$ and $\bar{\Gamma} = \frac{\Gamma}{K\langle A^0\rangle}$. Let $A_c/(2\langle A^0\rangle) = c$ and the value of A^0 in Eq. SM.15 be $\beta\langle A^0\rangle$. Then cells will grow if

$$\bar{\Lambda}s/2 + \bar{\Gamma}s^2\sqrt{c} < 2\sqrt{c}(\beta - c). \qquad \text{(SM.16)}$$

If the model of target area variation with the cell cycle and the critical area for cell division are fixed, then, for a given shape index (e.g. that of a regular hexagon), this forms a diagonal region in the phase space.

We note that equations SM.13 and SM.15 imply that cells with lower shape indices (i.e. more regular and with larger number of sides) are more likely to be stable.

In Fig. S10, we show the phase diagram in the space of the parameters $\bar{\Lambda}$ and $\bar{\Gamma}$. Region I is where cells are expected to have negative interfacial tensions and so have irregular shapes since they do not want to minimise their perimeters. The boundary of this domain is at $\Lambda + \Gamma s\sqrt{A^0} = 0$, so $\bar{\Lambda} + \bar{\Gamma}s = 0$. We plot this for hexagonal cells ($s = 2 \times 3^{1/4}2^{1/2}$). In Region II, cells take regular shapes and the only stable size is positive. The other boundary of this domain is at $\Lambda = 0$ or $\bar{\Lambda} = 0$. In Region III, cells take regular shapes and small cells collapse, whilst larger ones grow to an equilibrium size. In Region IV, all cells collapse. The boundary between these regions is given by $A^0 = \frac{\Gamma s^2}{2K} + \frac{3}{4}\left(\frac{\Lambda s}{K}\right)^{2/3}$ or $2 = \bar{\Gamma}s^2 + \frac{3}{2}(s\bar{\Lambda})^{2/3}$. We plot this for hexagonal cells. We also show as the dotted line, a line below which all cells should grow (Eq. SM.16). Here $\beta = 0.68/1.25$ is the minimum value of A^0 divided by its mean value. The minimal value is calculated from Eq. (1), assuming mean growth rate, and $c = 1.3/(2 \cdot 1.25)$ is the critical area divided by twice the mean value of A^0 (all nondimensional units).

III. TISSUE ASPECT RATIO DEPENDS ON EXPANSION RATE, THE VALUE OF Γ AND ON HOW EFFICIENTLY CELLS REARRANGE

To gain insight into the anisotropic growth of the tissue, we consider a slightly simpler system. We assume all cells are identical with area A and we assume that $\Lambda/\Gamma << 1$, so that the target perimeter is negligible. We assume cell division is unorientated. Let the dimensions of the neural tube be $R(t)$ and $H(t)$ and the number of cells be $N_c(t)$. Therefore $N_c A = RH$. For ease of analysis, we assume that cells are rectangular. In the direction of R, there are n_1 cells of length l_1, with $R = n_1 l_1$ and similarly in the other direction there are n_2 cells of length l_2. Therefore, $H/R = (n_2\, l_2)/(n_1\, l_1)$ and $N_c = n_1 n_2$. We start with an equal number of square cells in each direction. We consider two extreme cases. In the first case, intercalation can take place rapidly, allowing the number of cells in each direction to change. In this case, any change in the tissue shape and the dissipation of drag

Development • Supplementary information

forces will occur predominantly by changing the number of cells in each direction, rather than by altering cell shape. In such a scenario, the shape of individual cells will be relatively unaffected by anisotropic forces and will be square, hence $n_2/n_1 = H/R$. In the second case, intercalation does not take place, in which case as the aspect ratio of the whole tissue changes, the cells change shape accordingly. In this case, the relative number of cells in each direction does not change, since cell division is unorientated and intercalation impossible, hence $n_1 = n_2$ and $l_2/l_1 = H/R$.

The expansion of the tissue in dimensions R and H and perimeter $L = 2(l_1 + l_2)$, is given by SM.3. On the shortest timescales, we assume only l_1 and l_2 change. Therefore

$$\begin{aligned}\partial E/\partial R &= N_c K(A - A_0)\partial A/\partial R + N_c \Gamma L \partial L/\partial R \\ &= HK(A - A_0) + 2N_c \Gamma L/n_1 \\ &= HK(RH/N_c - A_0) + 4n_2\Gamma(l_1 + l_2) \\ &= H[K(RH/N_c - A_0) + 4\Gamma(1 + l_1/l_2)].\end{aligned} \tag{SM.17}$$

Similarly,

$$\partial E/\partial H = R\left[K(RH/N_c - A_0) + 4\Gamma(1 + l_2/l_1)\right]. \tag{SM.18}$$

In the first case, $\mu'/\mu''\, dR/dH = H/R$ and the change in R^2 will be μ''/μ' times the change in H^2, so the aspect ratio, H/R, will tend to $\sqrt{\frac{\mu'}{\mu''}}$.

In the second case,

$$\begin{aligned}\mu' N_c \frac{dR}{dt} &= H[K(A_0 - RH/N_c) - 4\Gamma(1 + R/H)] \\ \mu'' N_c \frac{dH}{dt} &= R[K(A_0 - RH/N_c) - 4\Gamma(1 + H/R)].\end{aligned} \tag{SM.19}$$

We expect that in the long term the growth will equilibrate so that the average areas and aspect ratios of cells will tend to constants, which we call $r = H/R$ and $A = RH/N_c$. Then we have that the length and height of the tissue satisfy

$$\begin{aligned}R &= \sqrt{A/r}\sqrt{N_c} \\ H &= \sqrt{rA}\sqrt{N_c}.\end{aligned}$$

If we assume that cell numbers grow exponentially with rate λ, so that $\frac{dN_c}{dt} = \lambda N_c$, then substituting into Eqs. SM.19 gives

$$\begin{aligned}\frac{\lambda}{2}\sqrt{N_c}\mu'\sqrt{A/r} &= \sqrt{rA}\sqrt{N_c}[K(A_0 - A) - 4\Gamma(1 + 1/r)] \\ \frac{\lambda}{2}\sqrt{N_c}\mu''\sqrt{rA} &= \sqrt{A/r}\sqrt{N_c}[K(A_0 - A) - 4\Gamma(1 + r)].\end{aligned}$$

Dividing by $\sqrt{N_c A}$ yields simultaneous equations for r and $A_0 - A$ with solution

$$\begin{aligned}r &= \sqrt{\frac{\mu'\lambda + 8\Gamma}{\mu''\lambda + 8\Gamma}} \\ A_0 - A &= \frac{8\Gamma + \sqrt{(\mu'\lambda + 8\Gamma)(\mu''\lambda + 8\Gamma)}}{2K}.\end{aligned}$$

This means that when growth is slow so that $\mu'\lambda, \mu''\lambda << 8\Gamma$, as $t \to \infty$, the aspect ratio should tend to one. When growth is fast so that $\mu''\lambda, \mu'\lambda >> 8\Gamma$, the aspect ratio should tend to $\sqrt{\frac{\mu'}{\mu''}}$. As the exponential growth rate of the tissue varies from small to large, the asymptotic aspect ratio will vary monotonically between these two values. Slow tissue growth is defined by the regime in which perimeter forces greatly exceed drag forces and rapid tissue growth by the regime in which drag forces greatly exceed perimeter forces. Cell pressure due to area elasticity is always important. In slow growth, pressure generated by the difference between target area and mean area is balanced by the stretched actomyosin ring, whereas in rapid growth it is balanced by drag.

The most extreme aspect ratios are achieved when the actomyosin ring exerts a negligible force or when tissue rearrangement is extremely efficient.

Farhadifar, R., Röper, J.-C., Aigouy, B., Eaton, S. and Jülicher, F. (2007). The influence of cell mechanics, cell-cell interactions, and proliferation on epithelial packing. *Current Biology* **17**, 2095 – 2104. ISSN 0960-9822.

Magno, R., Grieneisen, V. A. and Marée, A. F. (2015). The biophysical nature of cells: potential cell behaviours revealed by analytical and computational studies of cell surface mechanics. *BMC Biophysics* **8**, 8. ISSN 2046-1682.

Development • Supplementary information

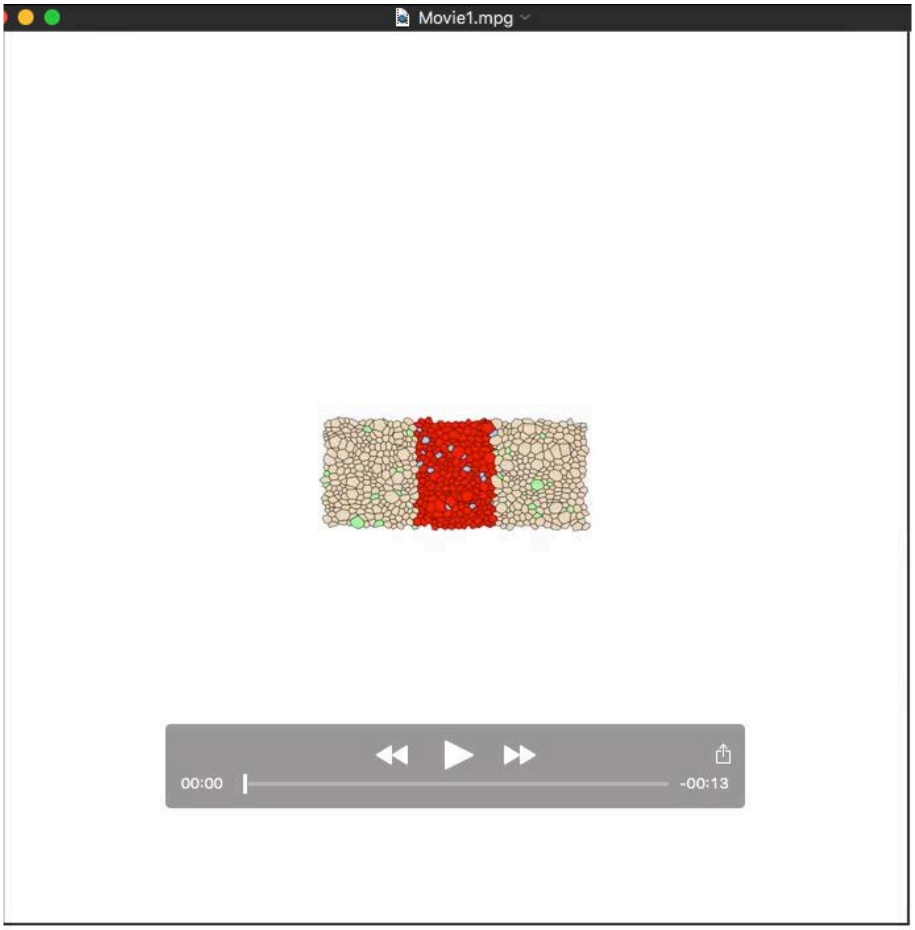

Movie 1. Simulation of clones tracked during the 48 h in a tissue with pD (brown) and pMN (red) populations. Green and blue cells represent clones in pD and pMN, respectively.
